# Early innate immune response triggered by the human respiratory syncytial virus and its regulation by ubiquitination/deubiquitination processes

**DOI:** 10.1186/s12929-022-00793-3

**Published:** 2022-02-13

**Authors:** María Martín-Vicente, Salvador Resino, Isidoro Martínez

**Affiliations:** 1 Unidad de Infección Viral E Inmunidad, Centro Nacional de Microbiología, Instituto de Salud Carlos III (Campus Majadahonda), Carretera Majadahonda-Pozuelo, Km 2.2, 28220 Majadahonda, Madrid, Spain; 2 Centro de Investigación Biomédica en Red en Enfermedades Infecciosas, Instituto de Salud Carlos III, Madrid, Spain

**Keywords:** Ubiquitination, Innate immunity, Immune Response Regulation, Respiratory Syncytial Virus

## Abstract

The human respiratory syncytial virus (HRSV) causes severe lower respiratory tract infections in infants and the elderly. An exuberant inadequate immune response is behind most of the pathology caused by the HRSV. The main targets of HRSV infection are the epithelial cells of the respiratory tract, where the immune response against the virus begins. This early innate immune response consists of the expression of hundreds of pro-inflammatory and anti-viral genes that stimulates subsequent innate and adaptive immunity. The early innate response in infected cells is mediated by intracellular signaling pathways composed of pattern recognition receptors (PRRs), adapters, kinases, and transcriptions factors. These pathways are tightly regulated by complex networks of post-translational modifications, including ubiquitination. Numerous ubiquitinases and deubiquitinases make these modifications reversible and highly dynamic. The intricate nature of the signaling pathways and their regulation offers the opportunity for fine-tuning the innate immune response against HRSV to control virus replication and immunopathology.

## Introduction

Human Respiratory Syncytial Virus (HRSV) is the leading cause of severe lower respiratory tract infections such as bronchiolitis and pneumonia in infants [17]. It also produces severe infections in the elderly and immunocompromised adults [36]. Worldwide, HRSV causes more than 33 million infections in children under five per year, of which approximately 3 million require hospital admission, and about 60.000 die [119, 150]. Bronchiolitis caused by HRSV infection is characterized by inflammation of the bronchial tubes and bronchioles of infected patients. Together with mucus, this inflammation obstructs the airway lumen reducing airflow through the airways. In the infant population, the airways are narrower, so they become more easily blocked, increasing the disease severity [135].

HRSV belongs to the *Orthopneumovirus* genus within the *Pneumoviridae* family. It is an enveloped virus with a non-segmented, single-stranded, negative RNA genome [2]. The HRSV genome contains ten genes that codify for 11 proteins. These include the attachment glycoprotein (G), the fusion protein (F), and the small hydrophobic (SH) protein, which are located on the virus surface. The nucleoprotein (N), phosphoprotein (P), large polymerase protein (L), M2 protein, and the matrix (M) protein are all placed inside the virion. Finally, the virus has two nonstructural (NS1 and NS2) proteins [25, 53].

### The early immune response against HRSV

The immune response against HRSV begins in the epithelial cells from respiratory airways, the main targets of virus infection. These cells produce multiple cytokines and chemokines (including CCL2, CCL3, CCL5, CCL7, CXCL10, CXCL11, IL-8, and IL-15) that trigger a pro-inflammatory/anti-viral response essential for virus control [111, 168, 192]. Conversely, the pro-inflammatory response also plays a prominent role in the pathogenesis of the disease [9, 11, 28, 56, 118, 136].

**Fig. 1:**
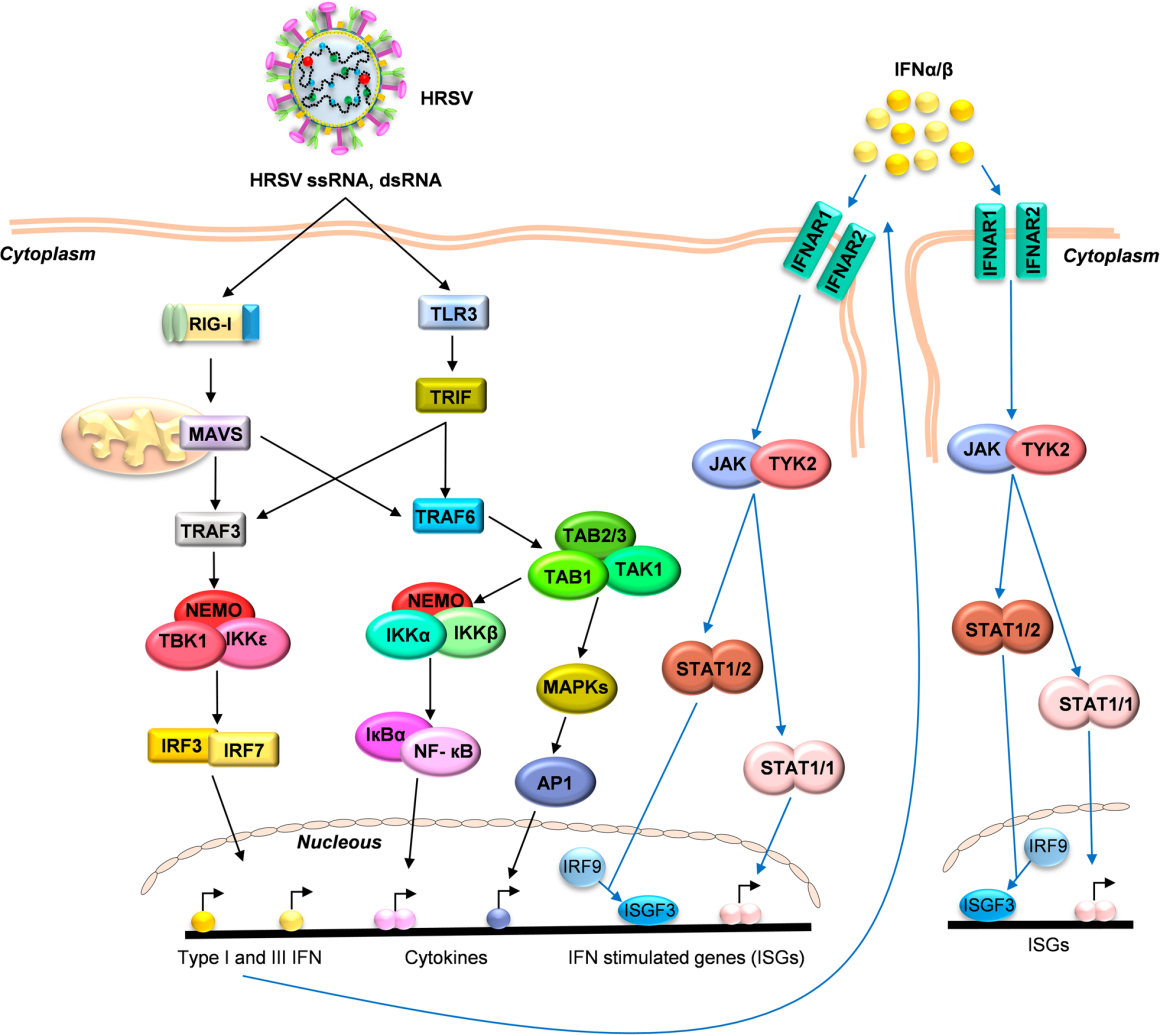
Main pathways activated in early innate anti-viral immunity after HRSV infection. The signaling pathways begin with the recognition of HRSV RNA by RIG-I and TLR3 receptors. The signal is transduced through adaptor proteins (MAVS, TRIF) to TRAF proteins (TRAF3/6), which activate the kinase complexes (NEMO, TBK1, IKKα/β/ε, TAB1, and TAK1), triggering the activation of transcription factors IRF3/7, NF-κB and AP1 to express type I/III IFN, cytokines, and anti-viral genes. The released IFNs binds to their receptors (IFNAR1/IFNAR2) in an autocrine and paracrine manner to induce JAK/STAT-mediated expression of multiple ISGs

The HRSV infection is detected in epithelial cells by Pattern Recognition Receptors (PRR) that recognize Pathogen-Associated Molecular Patterns (PAMPs), which, in HRSV infection, are mainly viral RNAs with 5’-triphosphate end and the double-strand RNA (dsRNA) produced during viral replication [52, 74, 96]. Those cell receptors trigger intracellular signaling involving different adaptors, kinases, and transcription factors (Fig. 1). The main PRRs that detect HRSV infection in epithelial cells are RIG-I Like Receptors (RLRs) and Toll-Like Receptors (TLRs) [34, 63, 78, 96, 107, 146]. These PRRs activate the transcription factors nuclear factor-κB (NF-κB) and interferon regulatory factors 3 and 7 (IRF3/7) (Fig. 1). The activation and translocation of these transcription factors to the cell nucleus triggers the expression of type I interferon (IFN-I), cytokines, chemokines, and anti-viral molecules [59, 73, 96, 112, 148].

#### RIG-I mediated signaling

RIG-I (Retinoic acid Inducible Gene I), a member of the RLR family, is the principal PRR involved in HRSV recognition in respiratory epithelial cells [96, 110, 183]. RIG-I silencing by siRNA inhibited the activation of both NF-κB and IRF3 transcription factors and the expression of IFN-β, CXCL10, CCL5, ISG15, TNF-α, and IL-6 at early times after HRSV infection [96, 110]. RIG-I comprises two N-terminal Caspases Activation and Recruitment Domains (CARDs), a central DExD/H box RNA helicase domain, and a regulatory C-Terminal Domain (CTD). The receptor is localized in the cell cytoplasm and, after viral infection, recognizes the 5’-triphosphate ends from single or double-strand viral RNAs [27, 143, 161, 184]. The viral RNA recognition induces a conformational change in RIG-I, leading to interaction with Mitochondrial Antiviral-Signaling proteins (MAVS, also known as Cardif, IPS1, or VISA). MAVS mediates the expression of most of the HRSV-induced genes in the lungs of infected mice, including IFN-I, IL-6, IL-1β, TNF-α, CCL2, CXCL1, and CXCL2 [12, 82, 129]. MAVS proteins contain TRAF-interacting motifs to interact with TRAF family proteins such as TRAF3/6 (TNF Receptor Associated Factor 3 and 6). This interaction leads to kinase-mediated activation of the transcription factors IRF3/7 and NF-κB and the subsequent expression of type I/III IFN, cytokines, chemokines, and anti-viral proteins [7, 10, 12–15, 23, 38, 40, 47, 52, 54, 58, 97, 98, 101, 113–115, 139, 140, 155, 159, 164–168, 183] (Fig. 1).

#### TLR3 mediated signaling

TLRs are among the best-characterized families that detect PAMPs from extracellular media, intracellular endosomes, and lysosomes [162]. In HRSV infection, the RNA recognition by TLR3 triggers the TRAF3/6-mediated signaling pathway and the transcription of several inflammatory and anti-viral immune response genes [55, 95, 96, 121, 141, 162] (Fig. 1). Following HRSV infection of airway epithelial cells, TLR3 is induced by RIG-I-dependent IFN-β secretion, indicating that RIG-I and TLR3 mediate the HRSV-induced innate immune response at different times postinfection [96].

#### IFN-mediated signaling

HRSV NS1 and NS2 proteins suppress type I IFN production [159]. Therefore, a robust IFN response is not observed in nasal secretions of HRSV infected infants [117]. However, type I and III interferons are produced in epithelial cells following HRSV-mediated RIG-I activation [96, 138, 185]. The transcription factors mediating IFN production are IRF3/7, activated by the kinase complex TBK1/IKKε/NEMO [39, 57, 99] (Fig. 1). The secreted IFN can act in a paracrine or autocrine manner by binding to its receptor (IFNAR), activating intracellular signaling pathways and leading to the expression of IFN-stimulated genes (ISGs). The IFNAR receptor is a cell surface transmembrane receptor composed of two subunits; IFNAR1 (IFN-α receptor 1) and IFNAR2. Both are associated with cytoplasmic tyrosine kinase 2 (Tyk2) and Janus-activated kinase 1 (Jak1). After IFN interaction, the tyrosine residues in the IFNAR cytoplasmic domains are phosphorylated to recruit and phosphorylate the “Signal Transducers and Activators of Transcription 1 and 2” (STAT1 and STAT2), leading to the formation of STAT1/STAT2 heterodimers and STAT1/STAT1 homodimers [49, 60, 83, 158] (Fig. 1). The STAT1 homodimers translocate to the nucleus, binding to IFN-gamma-activated sequences (GAS) sites in gene promoters and activating its transcription. On the other hand, STAT1/STAT2 heterodimers interact with IRF9 to form the ISGF3 (Interferon Stimulated Gene Factor 3) complex that binds to the interferon-stimulated response elements (ISRE) site, leading to the transcription of many ISGs, and establishing an anti-viral state [41, 89, 156].

In HRSV infection, IFN signaling plays an essential role in inducing pro-inflammatory cytokines and anti-viral genes [75, 83]. Goritzka et al. described the critical role of IFNAR in the innate resistance to HRSV infection in mice [50]. IFNAR1 deficient mice showed increased viral load and reduced type I/II/III IFN, pro-inflammatory cytokines, and chemokines in the lung in response to HRSV infection, indicating that signaling through IFNAR is necessary for coordinating the inflammatory response against HRSV [50]. Makris et al. reported that cytokine production is abolished in alveolar macrophages (AM) from IFNAR deficient mice infected with HRSV [106]. These data showed that the IFN pathway is critical for the innate immune response following HRSV infection. Therefore, inhibition of IFN signaling may help reduce inflammation in HRSV infections. However, the IFN pathway is also necessary to inhibit virus replication, so a fine-tune control of the innate immune response should be done to avoid immunopathological damage while restricting viral replication.

### The ubiquitination process

**Fig. 2:**
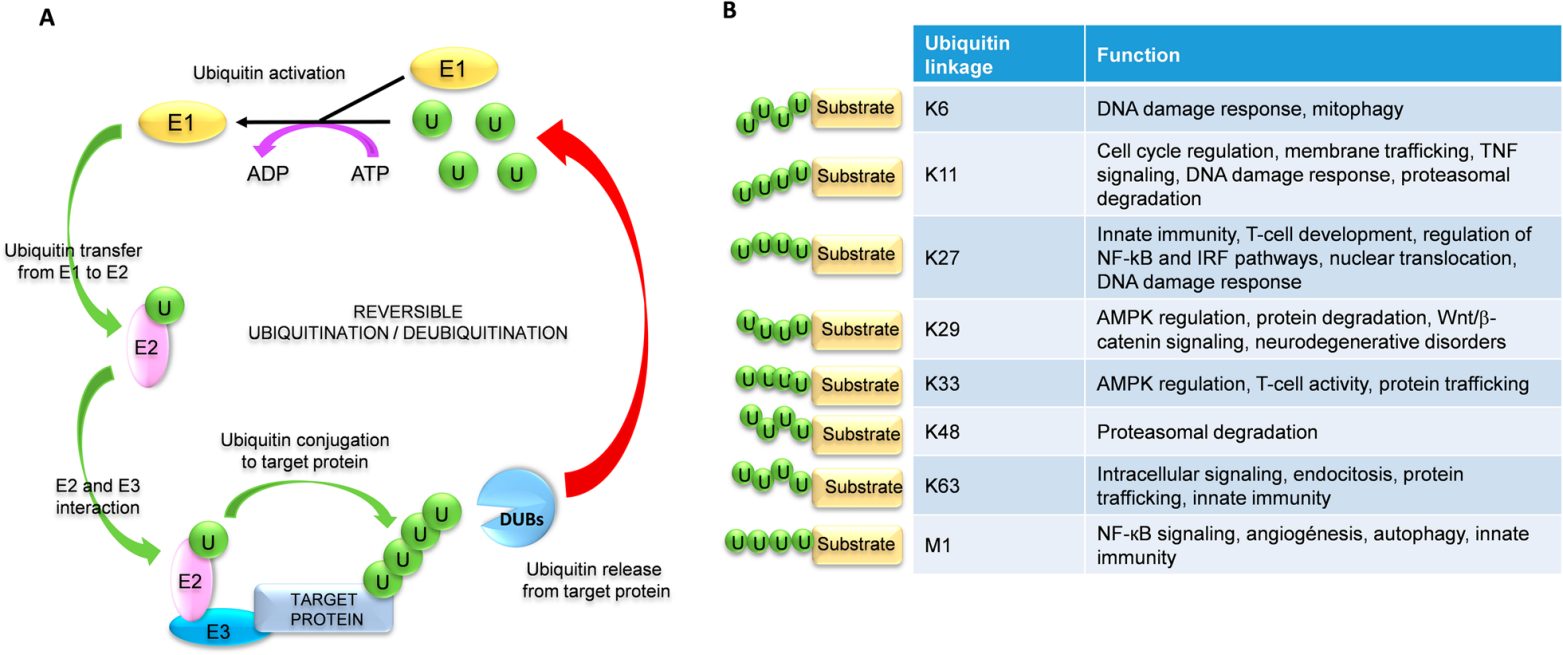
Ubiquitination/deubiquitination mechanisms. **A** Ubiquitination is a reversible three-step enzymatic process in which participate ubiquitin-activating (E1), ubiquitin-conjugating (E2), and ubiquitin ligase (E3) enzymes. Conjugated ubiquitins are removed from the target proteins by deubiquitinases (DUBs) (see text for full description). **B** Eight main types of ubiquitin chains with distinct cellular functions can be formed, depending on the ubiquitin lysine residue involved (K6, K11, K27, K29, K33, K48, or K63). Linear ubiquitin chains (M1) are formed by a head-to-tail linkage between the C-terminal carboxyl group of one ubiquitin and the N-terminal methionine of another ubiquitin molecule (see text for full description). A “U” inside a green circle depicts ubiquitin residues

The ubiquitin is a small protein of 76 amino acids conserved among different eukaryotic organisms. Ubiquitin can be conjugated to other proteins by covalent attachment between its C-terminal di-glycine motif and lysine (K) residues in the target protein. This covalent attachment, known as ubiquitination, modifies the activity or functionality of the target protein [6, 29, 134]. Ubiquitination is a three-step enzymatic process; firstly, the ubiquitin-activating enzyme (E1) activates the ubiquitin molecule in an ATP-dependent process. Secondly, the E1 protein transfers the activated ubiquitin to the ubiquitin-conjugating enzyme (E2). Lastly, the ubiquitin ligase (E3) interacts with E2 to attach the activated ubiquitin to the target protein through an isopeptide bond [24, 64, 81, 182] (Fig. 2A). The E3 enzyme determines the specificity of the target protein [29, 105, 197].

One (monoubiquitination), two (diubiquitination), or several (polyubiquitination) ubiquitin molecules can be attached to the target protein. The links between different ubiquitin molecules are also formed through covalent attachment of the C-Terminal di-glycine motif of one ubiquitin and the epsilon amino lysine residue of a second ubiquitin. The ubiquitin molecule has seven internal lysines (K6, K11, K27, K29, K33, K48, and K63), and therefore, several different types of ubiquitin chains with distinct functions can be formed (Fig. 2B) [29, 70, 81, 116]. The best-characterized are: (i) Ubiquitin linkage through lysine at position 48 (K48), which labels the target protein for proteasome recognition and degradation. (ii) Ubiquitin linkage through K63 activates intracellular signaling pathways by stabilizing substrates or acting as a scaffold that facilitates the formation of an active signaling complex [29, 200]. (iii) The linear ubiquitin chains are formed by covalent bonding between the C-terminal carboxyl group of one ubiquitin and the N-terminal methionine of another ubiquitin molecule [29].

Ubiquitination is a reversible process. Ubiquitin molecules can be removed from the target proteins by deubiquitinases (DUBs), which have protease and metalloprotease activity. The ubiquitin molecules are released to the cytosol for recycling or degraded in the proteasome [29] (Fig. 2A).

### Innate immune response regulation by ubiquitination/deubiquitination

In viral infections, the intracellular pathways activated by PRRs are tightly regulated by ubiquitination/deubiquitination and phosphorylation processes. These post-translational modifications modulate the activity, stability, or location of proteins involved in signaling pathways to ensure a proper immune/anti-viral response [57, 79, 108, 131, 142, 179] (Table 1).

**Table 1:** Ubiquitinases (E3 ligases) and deubiquitinases (proteases) that regulate intracellular signaling

Target protein	Enzyme (symbol)	Name	Activity	Ubiquitin linkage	Refs.
RIG-I	TRIM25	Tripartite Motif-containing protein 25	E3 ligase	K63	[44]
	RNF135	RING Finger Protein 135	E3 ligase	K63	[122]
	TRIM4	Tripartite Motif-containing protein 4	E3 ligase	K63	[181]
	MEX3C	Mex-3 RNA binding family member C	E3 ligase	K63	[84]
	USP4	Ubiquitin Specific Protease 4	Protease	K48	[172]
	RNF122	RING Finger Protein 122	E3 ligase	K48	[174]
	RNF125	RING Finger Protein 125	E3 ligase	K48	[5]
	CBL (c-Cbl)	Casitas B lineage lymphoma	E3 ligase	K48	[19]
	TRIM40	Tripartite Motif-containing protein 40	E3 ligase	K27 and K48	[193]
	CYLD	Cylindromatosis	Protease	K63	[42]
	USP21	Ubiquitin Specific Protease 21	Protease	K63	[37]
MAVS	TRIM31	Tripartite Motif-containing protein 31	E3 ligase	K63	[93]
	TRIM21	Tripartite Motif-containing protein 21	E3 ligase	K27	[180]
	RNF125	RING Finger Protein 125	E3 ligase	K48	[5]
	SMURF1/2	SMAD Specific E3 ubiquitin-protein ligase 1/2	E3 ligase	K48	[126, 176]
	ITCH	Itchy E3 ubiquitin-protein ligase	E3 ligase	K48	[22, 187]
	RNF5	RING Finger Protein 5	E3 ligase	K48	[199]
	MARCH5	Membrane-associated RING-CH 5	E3 ligase	K48	[186]
	TRIM25	Tripartite Motif-containing protein 25	E3 ligase	K48	[16]
	MARCH8	Membrane-associated RING-CH 8	E3 ligase	K27	[77]
	TRIM29	Tripartite Motif-containing protein 29	E3 ligase	K11	[178]
	YOD1 (OTUD2)	Ovarian Tumor Deubiquitinase2	Protease	K63	[94]
TRAF3	RNF166	RING Finger Protein 166	E3 ligase	K63	[18]
	HECTD3	HECT Domain E3 Ubiquitin Protein Ligase 3	E3 ligase	K63	[86]
	RNF216 (Triad3A)	RING Finger Protein 216	E3 ligase	K48	[120]
	OTUB1/2	OTU Deubiquitinase, Ubiquitin Aldehyde Binding 1 and 2	Protease	K63	[88]
	ZC3H12A (MCPIP1)	Monocyte Chemotactic Protein-Induced Protein-1	Protease	K63	[20, 91]
TRAF6	TRAF6	TNF Receptor Associated Factor 6	E3 ligase	K63	[85]
	RNF166	RING Finger Protein 166	E3 ligase	K63	[18]
	ZC3H12A (MCPIP1)	Monocyte Chemotactic Protein-Induced Protein-1	Protease	K63	[91]
	OTUB1/2	OTU Deubiquitinase, Ubiquitin Aldehyde Binding 1 and 2	Protease	K63	[88]
	TNFAIP3 (A20)	TNFα Induced Protein 3	Protease	K63	[3, 130, 149]
	TRIM38	Tripartite Motif-containing protein 38	E3 ligase	K48	[195]
TBK1	MIB1/2	MIB E3 ubiquitin-protein ligase	E3 ligase	K63	[87]
	RNF41 (Nrdp1)	RING Finger Protein 41	E3 ligase	K63	[170]
	RNF128	RING Finger Protein 128	E3 ligase	K63	[152]
	DTX4	Deltex E3 Ubiquitin Ligase 4	E3 ligase	K48	[26]
	TRAIP (TRIP)	TRAF Interacting Protein	E3 ligase	K48	[191]
	TRIM27	Tripartite Motif-containing protein 27	E3 ligase	K48	[198]
NEMO	TRAF6	TNF Receptor Associated Factor 6	E3 ligase	K63	[31]
	TRIM23	Tripartite Motif-containing protein 23	E3 ligase	K27	[4]
	LUBAC	Linear Ubiquitin chain Assembly Complex	E3 ligase	M1	[169]
	TRAF7	TNF Receptor Associated Factor 7	E3 ligase	K29	[201]
	TRIM29	Tripartite Motif-containing protein 29	E3 ligase	K48	[177]
TAK1	TRAF6	TNF Receptor Associated Factor 6	E3 ligase	K63	[171]
	CYLD	Cylindromatosis	Protease	K63	[1]
	ITCH	Itchy E3 ubiquitin-protein ligase	Protease	K63	[1]
TBK1-IKK complex	TNFAIP3 (A20)	TNFα Induced Protein 3	Protease	K63	[46, 127, 144]
IRF3	TRIM26	Tripartite Motif-containing protein 26	E3 ligase	K48	[173]
	TRIM21	Tripartite Motif-containing protein 21	E3 ligase	K48	[66]
	RBCK1 (RNF54)	RING Finger Protein 54	E3 ligase	K48	[190]
	CBL (c-Cbl)	Casitas B lineage lymphoma	E3 ligase	K48	[196]
	UBE3C (RAUL)	Ubiquitin Protein Ligase E3C	E3 ligase	K48	[188]
IRF7	UBE3C (RAUL)	Ubiquitin Protein Ligase E3C	E3 ligase	K48	[188]
	TRIM21	Tripartite Motif-containing protein 21	E3 ligase	K48	[65]
NF-κB	MKRN2	Makorin Ring Finger Protein 2	E3 ligase	K48	[151]
	PDLIM1	PDZ And LIM Domain 1	E3 ligase	K48	[163]
	COMMD1/Cul2	Copper Metabolism Domain Containing 1/Cullin 2	E3 ligase	K48	[48]
	TRAF7	TNF Receptor Associated Factor 7	E3 ligase	K29	[201]
STAT1	RNF31	RING Finger Protein 31	E3 ligase	M1	[202]
	OTULIN	OTU deubiquitinase with Linear linkage specificity	Protease	M1	[202]

Ubiquitination/deubiquitination processes regulate many proteins involved in RIG-I and TLR3 signaling pathways. Different E3 ligases (add ubiquitin chains to the target protein) or proteases (remove the ubiquitin chains from the target protein) modify the activity, localization, or stability of the target proteins. E3 ligases add ubiquitin residues to the target proteins; proteases (deubiquitinases) remove ubiquitin residues from the target proteins

#### RIG-I

RIG-I receptor is tightly modulated by complex ubiquitination and deubiquitination processes [44, 122, 125, 133, 189]. Its activity is positively regulated by TRIM25 and RNF135 (also known as RIPLET or REUL). Both TRIM25 and RNF135 contain an N-terminal RING (Really Interesting New Gene) domain with E3 ligase activity and a C-terminal PRY-SPRY domain [44, 45, 62, 109, 122, 123, 125]. RIG-I recognizes the 5’-triphosphate ends from small uncapped viral RNAs by its CTD. This interaction induces a conformational change in RIG-I that exposes its CARDs domains to interact with TRIM25. Subsequently, TRIM25 activates RIG-I via K63 polyubiquitination, while RNF135 activates RIG-I by K63-linked ubiquitin chains on its CTD domain [21, 44, 67, 122, 124, 145, 189]. RIG-I polyubiquitination promotes its interaction with MAVS and triggers downstream intracellular inflammatory and anti-viral responses [43, 44, 80, 137, 179]. Two other ubiquitin ligases have been reported to polyubiquitinate CARDs on RIG-I, namely TRIM4 and MEX3C [84, 181]. TRIM4 belongs to the TRIM family and adds ubiquitin residues through K63 to one of the CARDs of RIG-I, contributing to the RIG-I activation [181]. K63-linked ubiquitin residues added by MEX3C on different CARDs lysines of RIG-I increase the type-I IFN induction [84]. Additionally, the deubiquitinase USP4 removes K48-linked polyubiquitin chains on CARDs of RIG-I, allowing signal transduction [172].

There are also negative regulators of RIG-I activity: (i) RNF125 and RNF122 belong to the RING domain and E3-ligase family proteins, which ubiquitinate the CTD and CARDs in RIG-I through K48, respectively [5, 105, 123, 174]. (ii) c-Cbl (CBL) also ubiquitinates RIG-I through K48 on its CTD domain [19]. (iii) TRIM40 ubiquitinates RIG-I through K27 and K48 at the first CARD domain. In all these cases, these types of ubiquitination induce RIG-I proteasomal degradation [193]. (iv) CYLD is a deubiquitinase of the USP family that removes the K63 ubiquitin residues from RIG-I, inhibiting the interaction between RIG-I and MAVS [42]. (v) USP21 deubiquitinates the K63-linked ubiquitin chains on CARDs of RIG-I anchored by TRIM25 and RNF135 [37]. In all instances, the result is the inhibition of the RIG-I-mediated intracellular signaling pathway.

In HRSV infection, the K63 ubiquitination of RIG-I by TRIM25 induces the innate signaling pathways [7, 44, 102, 110]. The carboxy-terminal SPRY domain of TRIM25 interacts with the N-terminal CARDs or RIG-I to ubiquitinate Lys 172 of RIG-I [44]. Moreover, the E3 ubiquitin ligase FBXW7 ubiquitinates and degrades the SHP2 protein disrupting the SHP2/c-Cbl complex that mediates RIG-I degradation [153]. In both cases, these modifications promote RIG-I-mediated signaling.

#### MAVS

In HRSV infection, levels of MAVS are increased and mediate signaling pathways triggering the innate and adaptative immune response [12, 30, 129]. Upon RIG-I activation, CARD-CARD interaction between RIG-I and mitochondria-anchored MAVS induces a conformational change in MAVS to form a prion-like structure that activates downstream signaling [68]. The prion-like structure of MAVS recruits different TRAFs proteins like TRAF3/6, which promotes the activation of: (i) TBK1 complex (TBK1, IKKε, and NEMO (IKKγ)) that induces the IRF3/7 phosphorylation and their translocation into the nucleus to induce the transcription of type I IFN genes [100, 142]. (ii) IKK complex (IKKα/β and NEMO) that activates the NF-κB transcription factor and production of pro-inflammatory cytokines [100, 104, 162] (Fig. 1).

Post-translational modifications tightly regulate the MAVS adaptor to ensure a proper immune response. TRIM31 and TRIM21 are TRIM family members with E3 ubiquitin ligase activity that have been proposed as positive regulators of MAVS. TRIM31 catalyzes the formation of the prion-like aggregates through K63 ubiquitination of MAVS [93]. TRIM21 ubiquitinates MAVS through K27, enhancing its interaction with TBK1. In both cases, IRF3 and NF-κB signaling pathways are activated [180].

Numerous E3 ubiquitin ligases mediate the K48-linked ubiquitination and subsequent proteasome degradation of MAVS: RNF125 [5], Smurf1/2 [126, 176], ITCH [22, 187], RNF5 [199], MARCH5 [186], and TRIM25 [16]. Moreover, other E3 ligases, TRIM29 and MARCH8, ubiquitinate MAVS through K11 and K27, promoting MAVS degradation by the proteasome and autophagy, respectively [77, 178]. Recently, it has been described that the deubiquitinase YOD1 (OTUD2) removes K63-linked ubiquitin chains on MAVS, thereby abolishing the formation of MAVS prion-like aggregates and attenuating downstream signaling [94].

#### TRAF

TRAF3 and TRAF6 are involved in inducing the early innate immune response against HRSV. Thus, TRAF3 seems to be relevant in the signaling pathways induced by HRSV, as indicated by the NS1 and NS2-induced decrease of TRAF3 levels in HRSV infections [160]. TRAF6 is required for RIG-I-mediated p65 phosphorylation and subsequent activation of NF-κB transcription factor [183].

TRAF3/6 belongs to the TRAF family of adapter proteins, characterized by one conserved TRAF domain at the C-terminal end necessary to interact with other proteins and one RING domain at its N-terminal end with E3 ubiquitin ligase activity [72, 131]. MAVS recruits TRAF3/6 proteins to activate intracellular signaling [100] (Fig. 1). TRAF3/6 interaction with MAVS induces TRAF3/6 K63-linked autoubiquitination and activation [72].

Following activation, TRAF3 recruits TBK1 and IKKγ/ε to phosphorylate IRF3 and IRF7. The phosphorylated transcription factors translocate to the nucleus to induce type I IFNs and ISGs expression [121, 142] (Fig. 1). TRAF3 activity is modified by ubiquitination and deubiquitination processes. The RNF166 and HECTD3 are E3 ubiquitin ligases that ubiquitinate and activate TRAF3 via K63, inducing the signaling pathway [18, 86]. In contrast, another E3 ubiquitin ligase, RNF216 (also known as Triad3A), adds K48-linked ubiquitin residues to TRAF3, inducing its proteasomal degradation and inhibiting signal transduction [120]. Finally, TRAF3 can be negatively regulated by MCPIP1 (also known as ZC3H12A), OTUB1, and OTUB2. The MCPIP1 protein, as well as OTUB1 and OTUB2, removes K63-linked ubiquitin moieties from TRAF3, inhibiting cell signaling [20, 88, 91].

TRAF6 E3 ligase activity mediates K63-linked polyubiquitination of its substrates, including itself and NEMO [31, 85, 171]. Ubiquitinated TRAF6 serves as a scaffold for the recruitment and activation of the TAK1/TAB1/TAB2/3 complex and subsequent NF-κB activation (Fig. 1) [31, 157, 194]. Like TRAF3, TRAF6 is positively regulated by RNF166-mediated K63 ubiquitination [18]. Conversely, TRAF6 is negatively regulated by some proteins, including MCPIP1 [91], OTUB1, OTUB2 [88], TNFAIP3, and TRIM38. TNFAIP3 (also known as A20) removes K63-linked ubiquitin residues on TRAF6, inhibiting its activity and subsequent signaling [3, 130, 149]. The E3 ubiquitin ligase TRIM38 negatively regulates TRAF6 through K48 ubiquitination and subsequent degradation by the proteasome [195].

#### IKK and IKK-related kinases

The IkappaB kinases (IKKs), IKKα and IKKβ, and the IKK-related kinases TBK1 (TANK Binding Kinase 1) and IKKε are the last proteins to transduce the RLR-signaling pathway upstream of the transcription factors (Fig. 1). Both types of kinases interact with NEMO (IKKγ), a scaffold protein essential in the RIG-I-MAVS-mediated response against HRSV in infected cells [98]. NEMO recruits TBK1 (TANK Binding Kinase 1) and IKKε to form a complex that phosphorylates IRF3/7. Phosphorylated IRF3/7 translocate to the nucleus to induce the expression of type I/III IFNs, pro-inflammatory cytokines, and chemokines [148, 194] (Fig. 1).

In the case of NF-κB, its p65 (RelA) and p50 subunits are retained in the cytosol by the IκBα protein (Inhibitor of NF-κB proteins). The complex formed by NEMO, IKKα, and IKKβ phosphorylates IκBα, triggering its ubiquitination and subsequent proteasome-dependent degradation. Degradation of IκBα leads to the release of NF-κB and its translocation to the nucleus to express pro-inflammatory cytokines [61].

In HRSV infections, IKKβ is required for p65 phosphorylation and subsequent NF-κB translocation to the nucleus [33, 183]. Moreover, Haeberle et al*.* have reported that IKKβ and NEMO association is critical for NF-κB-mediated chemokine expression and lung inflammation [58]. Interestingly, after HRSV infection, IKKε appears to mediate both IRF3 and NF-κB-dependent gene expression [8, 71, 160]. Additionally, a decrease of TBK1 has been observed after treating cells with a potential drug against HRSV, indicating that TBK1 may participate in the HRSV-mediated immune signaling [69]. Finally, inhibition of TAK1 expression reduces HRSV-induced NF-κB-dependent gene expression [33].

The activity of these kinases is regulated by ubiquitination and deubiquitination processes. TBK1 is activated by the E3 ubiquitin ligases Mib1/2 [87], Nrdp1 (also known as RNF41) [170], and RNF128 [152], all of them add K63-linked ubiquitin residues to TBK1, promoting the downstream signaling pathway. On the other hand, DTX4 [26], TRIP (also known as TRAIP) [191], and TRIM27 [198], ubiquitinate TBK1 via K48 and label the protein for proteasomal degradation, inhibiting the RLR-mediated signaling cascade.

The IKK complex is regulated at different steps. NEMO activity is positively regulated by TRAF6, which ubiquitinates NEMO through K63 linkages, promoting IKK complex activation and the subsequent phosphorylation of IRF3/7 and NF-κB transcription factors [31]. Other E3 ubiquitin ligases, such as TRIM23 and LUBAC, promote the NEMO activity by adding K27 and M1-linked ubiquitin chains, respectively [4, 169]. In contrast, TRAF7 and TRIM29 induce NEMO degradation through K29 and K48-linked ubiquitination, respectively [177, 201]. TAK1 is also positively regulated by TRAF6 through the same mechanism as NEMO [171] but is negatively regulated by CYLD- and ITCH-mediated K63 deubiquitination [1].

Both TBK1 and IKK complexes are negatively regulated by the A20 deubiquitinase, which removes K63-ubiquitin chains on those proteins. In this role, A20 cooperates with TAX1BP1 (Tax1 Binding Protein 1) and ABIN1 (A20-binding inhibitor of NF-κB activation, also known as TNIP1) to disrupt the TRAF3-TBK1-IKKε complex and inhibit the IRF3 activation [46, 127, 144]. In line with this, our group found that downregulation of A20, TAX1BP1, or ABIN1 in HRSV infection increased the early innate immune response and reduced virus production in epithelial cells [108]. Accordingly, enhanced expression of inflammatory and anti-viral cytokines has been observed in TAX1BP1 knockout mice infected with HRSV [32].

#### Transcription factors: IRF3/7 and NF-κB

The last step in the RLR-signaling pathway is the activation of the transcription factors IRF3/7 and NF-κB [39, 57, 61, 99]. In HRSV infection, these transcription factors have been implicated in the induction of several pro-inflammatory cytokines and chemokines [7, 10, 23, 33, 38, 40, 54, 59, 71, 92, 96, 97, 115, 128, 140, 155, 164, 166–168, 183].

IRF3 is negatively regulated by K48 ubiquitination that promotes its proteasomal degradation. The E3 ubiquitin ligases involved in this process are: TRIM26, TRIM21, RBCK1 (also known as RNF54), c-Cbl, and RAUL (also known as UBE3C) [66, 173, 188, 190, 196]. IRF7 is also modulated by the ubiquitin E3 ligases RAUL and TRIM21 through the same degradative mechanism [65, 188].

NF-κB is also regulated by ubiquitination. The p65 (RelA) subunit is negatively regulated through K48 and K29 ubiquitination mediated by MKRN2, PDLIM1, COMMD1/Cul2, and TRAF7. Except for TRAF7, which ubiquitinates p65 through K29, all other ubiquitin E3 ligases add K48 ubiquitin chains to p65. However, both K29 and K48 ubiquitination result in p65 degradation and, consequently, the inactivation of NF-κB transcription factor [48, 151, 163, 201].

#### Components of the IFN signaling pathway

The HRSV innate immune response in epithelial cells begins with RIG-I activation, leading to type I/III IFN and pro-inflammatory cytokines expression. IFNs from infected cells trigger additional signaling pathways in the same and neighboring cells. These pathways are also tightly modulated by ubiquitination and deubiquitination processes. Thus, linear ubiquitination of STAT1 by RNF31 (also known as HOIP, a part of the LUBAC complex) prevents its interaction with IFNAR2. Consequently, STAT1 is not phosphorylated, and the anti-viral type I IFN signaling is inhibited [202]. As a positive regulator, the deubiquitinase OTULIN specifically removes linear ubiquitin chains from STAT1, allowing its phosphorylation and activation [202].

The HRSV infection activates and modulates STAT signaling pathways and subsequent ISGs expression [60, 75, 83, 138]. Wang et al*.* observed that the inhibition of HRSV replication by JAK-STAT1/2 activation is partially mediated by TRIM22 expression [175].

### Regulation of ubiquitination processes by HRSV proteins

**Fig. 3:**
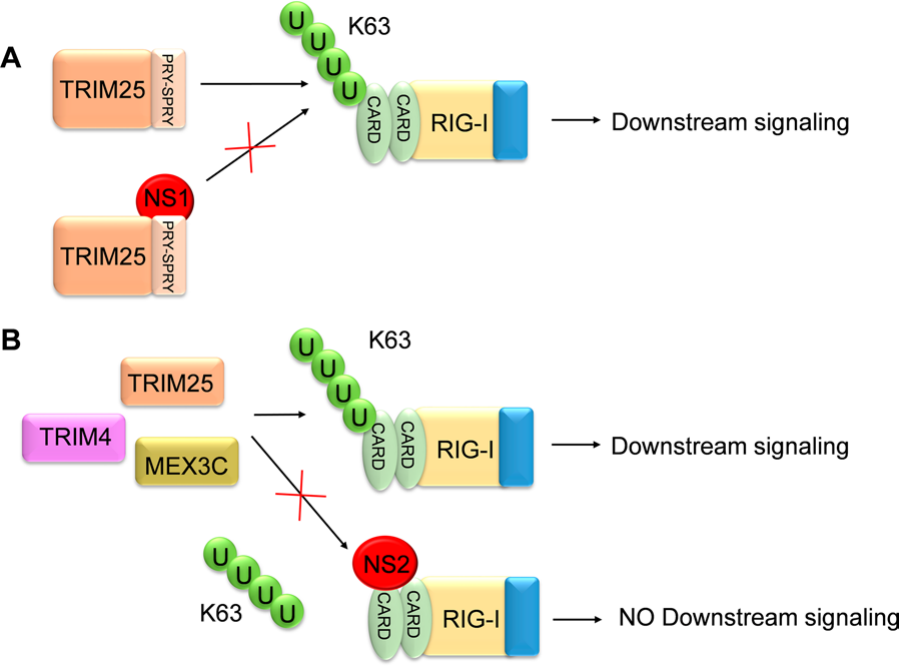
HRSV NS1 and NS2 inhibition of RIG-I ubiquitination. HRSV NS1 and NS2 proteins disrupt the innate immune cascade in infected cells by interfering with the K63 ubiquitination of RIG-I. This process involves NS1 binding to the PRY-SPRY motif of TRIM25 (**A**) or NS2 binding to RIG-I CARDs (**B**). A “U” inside a green circle depicts ubiquitin residues

HRSV counteracts the host’s innate immune response by different mechanisms. The viral proteins NS1 and NS2 play a crucial role in modulating RLR-mediated induction of type I and III IFN [76, 92, 103, 140, 147, 154]. The interaction between NS1 and TRIM25 is one of the best-known mechanisms to disrupting the RIG-I signaling pathway. The NS1 protein binds to the PRY-SPRY domain in TRIM25 to prevent K63 ubiquitination of RIG-I (Fig. 3A) and, consequently, its activation [7]. The HRSV NS2 protein also interferes with RIG-I activation by binding to the N-terminal CARD domain of RIG-I, preventing RNA recognition, ubiquitination of the domain, and its interaction with MAVS (Fig. 3B) [92, 132].

**Fig. 4:**
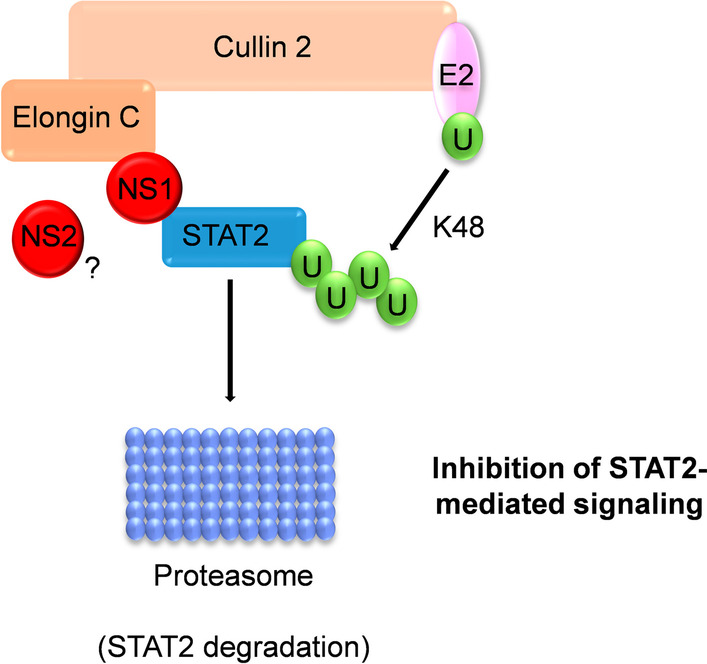
HRSV NS1-mediated STAT2 ubiquitination and degradation. NS1, and maybe NS2, form an E3 ligase complex with elongin C and cullin 2 that ubiquitinates STAT2 through K48 linkages. This modification labels STAT2 for proteasomal degradation, thus inhibiting IFN signaling. A “U” inside a green circle depicts ubiquitin residues

NS1 has a consensus sequence (**V**AL**L**KIT**C**YTDK) for binding to the elongin C and cullin 2 E3 ligase. Thus, it has been suggested that the NS1 may interact with elongin C and cullin 2 to form an E3 ligase complex that may ubiquitinate STAT2 for proteasomal degradation (Fig. 4) [35]. Although the NS2 protein does not appear to interact directly with the E3 ligase complex, it is necessary for effective STAT2 degradation, perhaps by bringing STAT2 closer to the NS1 E3 ligase complex or stabilizing or regulating the complex [35, 103]. However, Swedan et al*.* found a similar consensus sequence for potential binding to elongin C and cullin 2 in NS2 [160]. Therefore, the ability of NS2 to reduce STAT2 protein levels through a proteasomal mechanism may be mediated by this sequence (Fig. 4) [160].

Goswami et al*.* have described the so-called NS-degradosome (NSD), a large degradative complex containing the NS1 and NS2 proteins, as well as proteasomal and non-proteasomal proteases. Upon HRSV infection, NSD translocates to the mitochondria and interacts with MAVS. This association allows the degradation of several intermediates of the immune/anti-viral pathways, such as RIG-I, TRAF3, IKKε, or IRF3/7 [51]. However, it was not determined whether ubiquitination enzymes are structural components of the NSD or not.

**Fig. 5:**
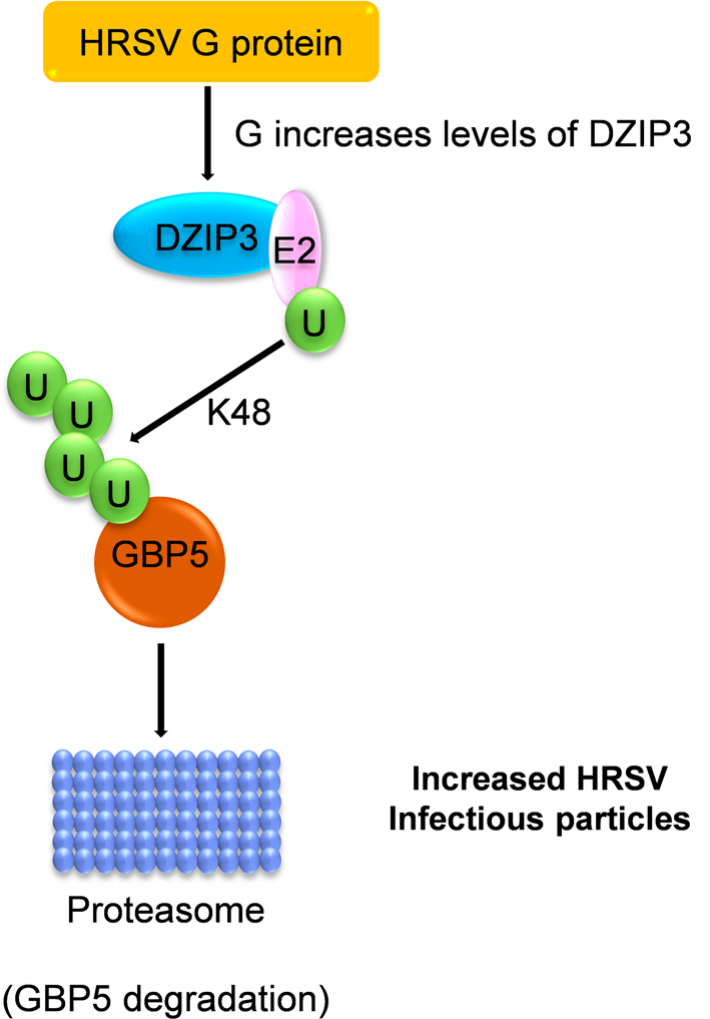
HRSV G-mediated ubiquitination and degradation of GBP5. HRSV glycoprotein G indirectly promotes GBP5 K48 ubiquitination for proteasomal degradation by raising the E3 ligase DZIP3 levels. Degradation of GBP5 increases the formation of HRSV infectious particles. A “U” inside a green circle depicts ubiquitin residues

Not only HRSV NS1 and NS2 proteins are involved in the regulation of the early innate immune response. GBP5 (Guanylate Binding Protein 5) is an IFNγ-inducible gene that belongs to the GTPase family and is involved in several cellular processes, such as inflammasome assembly, vesicle trafficking, and innate immunity. In HRSV infection, GBP5 reduces cell-associated SH protein levels by promoting SH release in cell culture, resulting in defective HRSV particles. However, HRSV modulates GBP5 expression in infected cells through the HRSV G protein. The G protein upregulates DZIP3 (DAZ Interacting Zinc Finger Protein 3), an E3 ligase that ubiquitinates GBP5 inducing its proteasomal degradation, promoting the generation of viable HRSV particles (Fig. 5) [90].

## Concluding remarks

Emerging data show that complex ubiquitination and deubiquitination processes are involved in the regulation of HRSV-induced early innate immunity. RIG-I, MAVS, TRAF3/6, and NEMO are the main proteins regulated by these processes. Ubiquitination/deubiquitination of K63 or K48-linked chains are the most frequent modifications.

Regulation of innate immune pathways in infected cells may impact HRSV dissemination and adaptative immunity. Therefore, proteins participating in those pathways are potential targets for controlling virus replication and immunopathology.

## Data Availability

Not applicable.

## References

[1] Ahmed N, Zeng M, Sinha I, Polin L, Wei WZ, Rathinam C, Flavell R, Massoumi R, Venuprasad K (2011). The E3 ligase Itch and deubiquitinase Cyld act together to regulate Tak1 and inflammation. Nat Immunol.

[2] Amarasinghe GK, Ayllon MA, Bao Y, Basler CF, Bavari S, Blasdell KR, Briese T, Brown PA, Bukreyev A, Balkema-Buschmann A, Buchholz UJ, Chabi-Jesus C, Chandran K, Chiapponi C, Crozier I, de Swart RL, Dietzgen RG, Dolnik O, Drexler JF, Durrwald R, Dundon WG, Duprex WP, Dye JM, Easton AJ, Fooks AR, Formenty PBH, Fouchier RAM, Freitas-Astua J, Griffiths A, Hewson R, Horie M, Hyndman TH, Jiang D, Kitajima EW, Kobinger GP, Kondo H, Kurath G, Kuzmin IV, Lamb RA, Lavazza A, Lee B, Lelli D, Leroy EM, Li J, Maes P, Marzano SL, Moreno A, Muhlberger E, Netesov SV, Nowotny N, Nylund A, Okland AL, Palacios G, Palyi B, Paweska JT, Payne SL, Prosperi A, Ramos-Gonzalez PL, Rima BK, Rota P, Rubbenstroth D, Shi M, Simmonds P, Smither SJ, Sozzi E, Spann K, Stenglein MD, Stone DM, Takada A, Tesh RB, Tomonaga K, Tordo N, Towner JS, van den Hoogen B, Vasilakis N, Wahl V, Walker PJ, Wang LF, Whitfield AE, Williams JV, Zerbini FM, Zhang T, Zhang YZ, Kuhn JH (2019). Taxonomy of the order Mononegavirales: update 2019. Adv Virol.

[3] Arguello M, Paz S, Ferran C, Moll HP, Hiscott J (2014). Anti-viral tetris: modulation of the innate anti-viral immune response by A20. Adv Exp Med Biol.

[4] Arimoto K, Funami K, Saeki Y, Tanaka K, Okawa K, Takeuchi O, Akira S, Murakami Y, Shimotohno K (2010). Polyubiquitin conjugation to NEMO by triparite motif protein 23 (TRIM23) is critical in antiviral defense. Proc Natl Acad Sci U S A.

[5] Arimoto K, Takahashi H, Hishiki T, Konishi H, Fujita T, Shimotohno K (2007). Negative regulation of the RIG-I signaling by the ubiquitin ligase RNF125. Proc Natl Acad Sci U S A.

[6] Baker RT, Board PG (1987). The human ubiquitin gene family: structure of a gene and pseudogenes from the Ub B subfamily. Nucleic Acids Res.

[7] Ban J, Lee NR, Lee NJ, Lee JK, Quan FS, Inn KS (2018). Human respiratory syncytial virus NS 1 targets TRIM25 to suppress RIG-I ubiquitination and subsequent RIG-I-mediated antiviral signaling. Viruses.

[8] Bao X, Indukuri H, Liu T, Liao SL, Tian B, Brasier AR, Garofalo RP, Casola A (2010). IKKepsilon modulates RSV-induced NF-kappaB-dependent gene transcription. Virology.

[9] Bermejo-Martin JF, Bernardo D, Dominguez-Gil M, Alonso A, Garcia-Arevalo MC, Pino M, de Lejarazu RO, Eiros JM, Ardura J, Leon AJ, Garrote JA, Resino S, Blanco-Quiros A, Munoz-Fernandez MA, Arranz E (2006). Interleukin (IL)-1beta, IL-6 and IL-8 in nasal secretions: a common role for innate immunity in viral bronchial infection in infants?. Br J Biomed Sci.

[10] Bertolusso R, Tian B, Zhao Y, Vergara L, Sabree A, Iwanaszko M, Lipniacki T, Brasier AR, Kimmel M (2014). Dynamic cross talk model of the epithelial innate immune response to double-stranded RNA stimulation: coordinated dynamics emerging from cell-level noise. PLoS ONE.

[11] Bertrand P, Lay MK, Piedimonte G, Brockmann PE, Palavecino CE, Hernandez J, Leon MA, Kalergis AM, Bueno SM (2015). Elevated IL-3 and IL-12p40 levels in the lower airway of infants with RSV-induced bronchiolitis correlate with recurrent wheezing. Cytokine.

[12] Bhoj VG, Sun Q, Bhoj EJ, Somers C, Chen X, Torres JP, Mejias A, Gomez AM, Jafri H, Ramilo O, Chen ZJ (2008). MAVS and MyD88 are essential for innate immunity but not cytotoxic T lymphocyte response against respiratory syncytial virus. Proc Natl Acad Sci U S A.

[13] Bitko V, Barik S (1998). Persistent activation of RelA by respiratory syncytial virus involves protein kinase C, underphosphorylated IkappaBbeta, and sequestration of protein phosphatase 2A by the viral phosphoprotein. J Virol.

[14] Bitko V, Velazquez A, Yang L, Yang YC, Barik S (1997). Transcriptional induction of multiple cytokines by human respiratory syncytial virus requires activation of NF-kappa B and is inhibited by sodium salicylate and aspirin. Virology.

[15] Carpenter LR, Moy JN, Roebuck KA (2002). Respiratory syncytial virus and TNF alpha induction of chemokine gene expression involves differential activation of Rel A and NF-kappa B1. BMC Infect Dis.

[16] Castanier C, Zemirli N, Portier A, Garcin D, Bidere N, Vazquez A, Arnoult D (2012). MAVS ubiquitination by the E3 ligase TRIM25 and degradation by the proteasome is involved in type I interferon production after activation of the antiviral RIG-I-like receptors. BMC Biol.

[17] Chanock R, Roizman B, Myers R (1957). Recovery from infants with respiratory illness of a virus related to chimpanzee coryza agent (CCA) I Isolation, properties and characterization. Am J Hyg.

[18] Chen HW, Yang YK, Xu H, Yang WW, Zhai ZH, Chen DY (2015). Ring finger protein 166 potentiates RNA virus-induced interferon-beta production via enhancing the ubiquitination of TRAF3 and TRAF6. Sci Rep.

[19] Chen W, Han C, Xie B, Hu X, Yu Q, Shi L, Wang Q, Li D, Wang J, Zheng P, Liu Y, Cao X (2013). Induction of Siglec-G by RNA viruses inhibits the innate immune response by promoting RIG-I degradation. Cell.

[20] Chen X, Zhao Q, Xie Q, Xing Y, Chen Z (2018). MCPIP1 negatively regulate cellular antiviral innate immune responses through DUB and disruption of TRAF3-TBK1-IKKepsilon complex. Biochem Biophys Res Commun.

[21] Chiang C, Gack MU (2017). Post-translational control of intracellular pathogen sensing pathways. Trends Immunol.

[22] Choi YB, Shembade N, Parvatiyar K, Balachandran S, Harhaj EW (2017). TAX1BP1 restrains virus-induced apoptosis by facilitating itch-mediated degradation of the mitochondrial adaptor MAVS. Mol Cell Biol.

[23] Choudhary S, Boldogh S, Garofalo R, Jamaluddin M, Brasier AR (2005). Respiratory syncytial virus influences NF-kappaB-dependent gene expression through a novel pathway involving MAP3K14/NIK expression and nuclear complex formation with NF-kappaB2. J Virol.

[24] Ciechanover A, Elias S, Heller H, Hershko A (1982). "Covalent affinity" purification of ubiquitin-activating enzyme. J Biol Chem.

[25] Collins PL, Dickens LE, Buckler-White A, Olmsted RA, Spriggs MK, Camargo E, Coelingh KV (1986). Nucleotide sequences for the gene junctions of human respiratory syncytial virus reveal distinctive features of intergenic structure and gene order. Proc Natl Acad Sci U S A.

[26] Cui J, Li Y, Zhu L, Liu D, Songyang Z, Wang HY, Wang RF (2012). NLRP4 negatively regulates type I interferon signaling by targeting the kinase TBK1 for degradation via the ubiquitin ligase DTX4. Nat Immunol.

[27] Cui S, Eisenacher K, Kirchhofer A, Brzozka K, Lammens A, Lammens K, Fujita T, Conzelmann KK, Krug A, Hopfner KP (2008). The C-terminal regulatory domain is the RNA 5'-triphosphate sensor of RIG-I. Mol Cell.

[28] Das S, Palmer OP, Leight WD, Surowitz JB, Pickles RJ, Randell SH, Buchman CA (2005). Cytokine amplification by respiratory syncytial virus infection in human nasal epithelial cells. Laryngoscope.

[29] Davis ME, Gack MU (2015). Ubiquitination in the antiviral immune response. Virology.

[30] Demoor T, Petersen BC, Morris S, Mukherjee S, Ptaschinski C, De Almeida Nagata DE, Kawai T, Ito T, Akira S, Kunkel SL, Schaller MA, Lukacs NW (2012). IPS-1 signaling has a nonredundant role in mediating antiviral responses and the clearance of respiratory syncytial virus. J Immunol.

[31] Deng L, Wang C, Spencer E, Yang L, Braun A, You J, Slaughter C, Pickart C, Chen ZJ (2000). Activation of the IkappaB kinase complex by TRAF6 requires a dimeric ubiquitin-conjugating enzyme complex and a unique polyubiquitin chain. Cell.

[32] Descamps D, PeresdeOliveira A, Gonnin L, Madrieres S, Fix J, Drajac C, Marquant Q, Bouguyon E, Pietralunga V, Iha H, MoraisVentura A, Tangy F, Vidalain PO, Eleouet JF, Galloux M (2021). Depletion of TAX1BP1 Amplifies Innate Immune Responses during Respiratory Syncytial Virus Infection. J Virol.

[33] Dey N, Liu T, Garofalo RP, Casola A (2011). TAK1 regulates NF-KappaB and AP-1 activation in airway epithelial cells following RSV infection. Virology.

[34] Dou Y, Zhao Y, Zhang ZY, Mao HW, Tu WW, Zhao XD (2013). Respiratory syncytial virus infection induces higher Toll-like receptor-3 expression and TNF-alpha production than human metapneumovirus infection. PLoS ONE.

[35] Elliott J, Lynch OT, Suessmuth Y, Qian P, Boyd CR, Burrows JF, Buick R, Stevenson NJ, Touzelet O, Gadina M, Power UF, Johnston JA (2007). Respiratory syncytial virus NS1 protein degrades STAT2 by using the Elongin-Cullin E3 ligase. J Virol.

[36] Falsey AR, Hennessey PA, Formica MA, Cox C, Walsh EE (2005). Respiratory syncytial virus infection in elderly and high-risk adults. N Engl J Med.

[37] Fan Y, Mao R, Yu Y, Liu S, Shi Z, Cheng J, Zhang H, An L, Zhao Y, Xu X, Chen Z, Kogiso M, Zhang D, Zhang H, Zhang P, Jung JU, Li X, Xu G, Yang J (2014). USP21 negatively regulates antiviral response by acting as a RIG-I deubiquitinase. J Exp Med.

[38] Fang L, Choudhary S, Tian B, Boldogh I, Yang C, Ivanciuc T, Ma Y, Garofalo RP, Brasier AR (2015). Ataxia telangiectasia mutated kinase mediates NF-kappaB serine 276 phosphorylation and interferon expression via the IRF7-RIG-I amplification loop in paramyxovirus infection. J Virol.

[39] Fang R, Wang C, Jiang Q, Lv M, Gao P, Yu X, Mu P, Zhang R, Bi S, Feng JM, Jiang Z (2017). NEMO-IKKbeta Are Essential for IRF3 and NF-kappaB Activation in the cGAS-STING Pathway. J Immunol.

[40] Fiedler MA, Wernke-Dollries K, Stark JM (1996). Inhibition of viral replication reverses respiratory syncytial virus-induced NF-kappaB activation and interleukin-8 gene expression in A549 cells. J Virol.

[41] Fink K, Grandvaux N (2013). STAT2 and IRF9: Beyond ISGF3. JAKSTAT.

[42] Friedman CS, O'Donnell MA, Legarda-Addison D, Ng A, Cardenas WB, Yount JS, Moran TM, Basler CF, Komuro A, Horvath CM, Xavier R, Ting AT (2008). The tumour suppressor CYLD is a negative regulator of RIG-I-mediated antiviral response. EMBO Rep.

[43] Gack MU, Kirchhofer A, Shin YC, Inn KS, Liang C, Cui S, Myong S, Ha T, Hopfner KP, Jung JU (2008). Roles of RIG-I N-terminal tandem CARD and splice variant in TRIM25-mediated antiviral signal transduction. Proc Natl Acad Sci U S A.

[44] Gack MU, Shin YC, Joo CH, Urano T, Liang C, Sun L, Takeuchi O, Akira S, Chen Z, Inoue S, Jung JU (2007). TRIM25 RING-finger E3 ubiquitin ligase is essential for RIG-I-mediated antiviral activity. Nature.

[45] Gao D, Yang YK, Wang RP, Zhou X, Diao FC, Li MD, Zhai ZH, Jiang ZF, Chen DY (2009). REUL is a novel E3 ubiquitin ligase and stimulator of retinoic-acid-inducible gene-I. PLoS ONE.

[46] Gao L, Coope H, Grant S, Ma A, Ley SC, Harhaj EW (2011). ABIN1 protein cooperates with TAX1BP1 and A20 proteins to inhibit antiviral signaling. J Biol Chem.

[47] Garofalo R, Sabry M, Jamaluddin M, Yu RK, Casola A, Ogra PL, Brasier AR (1996). Transcriptional activation of the interleukin-8 gene by respiratory syncytial virus infection in alveolar epithelial cells: nuclear translocation of the RelA transcription factor as a mechanism producing airway mucosal inflammation. J Virol.

[48] Geng H, Wittwer T, Dittrich-Breiholz O, Kracht M, Schmitz ML (2009). Phosphorylation of NF-kappaB p65 at Ser468 controls its COMMD1-dependent ubiquitination and target gene-specific proteasomal elimination. EMBO Rep.

[49] González-Sanz R, Mata M, Bermejo-Martín J, Álvarez A, Cortijo J, Melero JA, Martínez I (2016). ISG15 Is Upregulated in Respiratory Syncytial Virus Infection and Reduces Virus Growth through Protein ISGylation. J Virol.

[50] Goritzka M, Durant LR, Pereira C, Salek-Ardakani S, Openshaw PJ, Johansson C (2014). Alpha/beta interferon receptor signaling amplifies early proinflammatory cytokine production in the lung during respiratory syncytial virus infection. J Virol.

[51] Goswami R, Majumdar T, Dhar J, Chattopadhyay S, Bandyopadhyay SK, Verbovetskaya V, Sen GC, Barik S (2013). Viral degradasome hijacks mitochondria to suppress innate immunity. Cell Res.

[52] Goubau D, Schlee M, Deddouche S, Pruijssers AJ, Zillinger T, Goldeck M, Schuberth C, Van der Veen AG, Fujimura T, Rehwinkel J, Iskarpatyoti JA, Barchet W, Ludwig J, Dermody TS, Hartmann G, Reis e Sousa C (2014). Antiviral immunity via RIG-I-mediated recognition of RNA bearing 5'-diphosphates. Nature.

[53] Gould PS, Easton AJ (2007). Coupled translation of the second open reading frame of M2 mRNA is sequence dependent and differs significantly within the subfamily Pneumovirinae. J Virol.

[54] Grandvaux N, Guan X, Yoboua F, Zucchini N, Fink K, Doyon P, Martin L, Servant MJ, Chartier S (2014). Sustained activation of interferon regulatory factor 3 during infection by paramyxoviruses requires MDA5. J Innate Immun.

[55] Groskreutz DJ, Monick MM, Powers LS, Yarovinsky TO, Look DC, Hunninghake GW (2006). Respiratory syncytial virus induces TLR3 protein and protein kinase R, leading to increased double-stranded RNA responsiveness in airway epithelial cells. J Immunol.

[56] Guo-Parke H, Canning P, Douglas I, Villenave R, Heaney LG, Coyle PV, Lyons JD, Shields MD, Power UF (2013). Relative respiratory syncytial virus cytopathogenesis in upper and lower respiratory tract epithelium. Am J Respir Crit Care Med.

[57] Hacker H, Karin M (2006). Regulation and function of IKK and IKK-related kinases. Sci STKE.

[58] Haeberle HA, Casola A, Gatalica Z, Petronella S, Dieterich HJ, Ernst PB, Brasier AR, Garofalo RP (2004). IkappaB kinase is a critical regulator of chemokine expression and lung inflammation in respiratory syncytial virus infection. J Virol.

[59] Haeberle HA, Takizawa R, Casola A, Brasier AR, Dieterich HJ, Van Rooijen N, Gatalica Z, Garofalo RP (2002). Respiratory syncytial virus-induced activation of nuclear factor-kappaB in the lung involves alveolar macrophages and toll-like receptor 4-dependent pathways. J Infect Dis.

[60] Hashimoto K, Durbin JE, Zhou W, Collins RD, Ho SB, Kolls JK, Dubin PJ, Sheller JR, Goleniewska K, O'Neal JF, Olson SJ, Mitchell D, Graham BS, Peebles RS (2005). Respiratory syncytial virus infection in the absence of STAT 1 results in airway dysfunction, airway mucus, and augmented IL-17 levels. J Allergy Clin Immunol.

[61] Hayden MS, West AP, Ghosh S (2006). NF-kappaB and the immune response. Oncogene.

[62] Hayman TJ, Hsu AC, Kolesnik TB, Dagley LF, Willemsen J, Tate MD, Baker PJ, Kershaw NJ, Kedzierski L, Webb AI, Wark PA, Kedzierska K, Masters SL, Belz GT, Binder M, Hansbro PM, Nicola NA, Nicholson SE (2019). RIPLET, and not TRIM25, is required for endogenous RIG-I-dependent antiviral responses. Immunol Cell Biol.

[63] Haynes LM, Moore DD, Kurt-Jones EA, Finberg RW, Anderson LJ, Tripp RA (2001). Involvement of toll-like receptor 4 in innate immunity to respiratory syncytial virus. J Virol.

[64] Hershko A, Heller H, Elias S, Ciechanover A (1983). Components of ubiquitin-protein ligase system. Resolution, affinity purification, and role in protein breakdown. J Biol Chem.

[65] Higgs R, Lazzari E, Wynne C, Gabhann J, Espinosa A, Wahren-Herlenius M, Jefferies CA (2010). Self protection from anti-viral responses–Ro52 promotes degradation of the transcription factor IRF7 downstream of the viral Toll-Like receptors. PLoS ONE.

[66] Higgs R, Ni Gabhann J, Ben Larbi N, Breen EP, Fitzgerald KA, Jefferies CA (2008). The E3 ubiquitin ligase Ro52 negatively regulates IFN-beta production post-pathogen recognition by polyubiquitin-mediated degradation of IRF3. J Immunol.

[67] Hornung V, Ellegast J, Kim S, Brzozka K, Jung A, Kato H, Poeck H, Akira S, Conzelmann KK, Schlee M, Endres S, Hartmann G (2006). 5'-Triphosphate RNA is the ligand for RIG-I. Science.

[68] Hou F, Sun L, Zheng H, Skaug B, Jiang QX, Chen ZJ (2011). MAVS forms functional prion-like aggregates to activate and propagate antiviral innate immune response. Cell.

[69] Huo X, Hou D, Wang H, He B, Fang J, Meng Y, Liu L, Wei Z, Wang Z, Liu FW (2021). Design, synthesis, in vitro and in vivo anti-respiratory syncytial virus (RSV) activity of novel oxizine fused benzimidazole derivatives. Eur J Med Chem.

[70] Ikeda F, Crosetto N, Dikic I (2010). What determines the specificity and outcomes of ubiquitin signaling?. Cell.

[71] Indukuri H, Castro SM, Liao SM, Feeney LA, Dorsch M, Coyle AJ, Garofalo RP, Brasier AR, Casola A (2006). Ikkepsilon regulates viral-induced interferon regulatory factor-3 activation via a redox-sensitive pathway. Virology.

[72] Inoue J, Ishida T, Tsukamoto N, Kobayashi N, Naito A, Azuma S, Yamamoto T (2000). Tumor necrosis factor receptor-associated factor (TRAF) family: adapter proteins that mediate cytokine signaling. Exp Cell Res.

[73] Jamaluddin M, Casola A, Garofalo RP, Han Y, Elliott T, Ogra PL, Brasier AR (1998). The major component of IkappaBalpha proteolysis occurs independently of the proteasome pathway in respiratory syncytial virus-infected pulmonary epithelial cells. J Virol.

[74] Jiang F, Ramanathan A, Miller MT, Tang GQ, Gale M, Patel SS, Marcotrigiano J (2011). Structural basis of RNA recognition and activation by innate immune receptor RIG-I. Nature.

[75] Jie Z, Dinwiddie DL, Senft AP, Harrod KS (2011). Regulation of STAT signaling in mouse bone marrow derived dendritic cells by respiratory syncytial virus. Virus Res.

[76] Jin H, Zhou H, Cheng X, Tang R, Munoz M, Nguyen N (2000). Recombinant respiratory syncytial viruses with deletions in the NS1, NS2, SH, and M2–2 genes are attenuated in vitro and in vivo. Virology.

[77] Jin S, Tian S, Luo M, Xie W, Liu T, Duan T, Wu Y, Cui J (2017). Tetherin suppresses type i interferon signaling by targeting MAVS for NDP52-mediated selective autophagic degradation in human cells. Mol Cell.

[78] Johnson TR, Rao S, Seder RA, Chen M, Graham BS (2009). TLR9 agonist, but not TLR7/8, functions as an adjuvant to diminish FI-RSV vaccine-enhanced disease, while either agonist used as therapy during primary RSV infection increases disease severity. Vaccine.

[79] Kanarek N, Ben-Neriah Y (2012). Regulation of NF-kappaB by ubiquitination and degradation of the IkappaBs. Immunol Rev.

[80] Kawai T, Takahashi K, Sato S, Coban C, Kumar H, Kato H, Ishii KJ, Takeuchi O, Akira S (2005). IPS-1, an adaptor triggering RIG-I- and Mda5-mediated type I interferon induction. Nat Immunol.

[81] Kerscher O, Felberbaum R, Hochstrasser M (2006). Modification of proteins by ubiquitin and ubiquitin-like proteins. Annu Rev Cell Dev Biol.

[82] Kirsebom FCM, Kausar F, Nuriev R, Makris S, Johansson C (2019). Neutrophil recruitment and activation are differentially dependent on MyD88/TRIF and MAVS signaling during RSV infection. Mucosal Immunol.

[83] Kong X, San Juan H, Kumar M, Behera AK, Mohapatra A, Hellermann GR, Mane S, Lockey RF, Mohapatra SS (2003). Respiratory syncytial virus infection activates STAT signaling in human epithelial cells. Biochem Biophys Res Commun.

[84] Kuniyoshi K, Takeuchi O, Pandey S, Satoh T, Iwasaki H, Akira S, Kawai T (2014). Pivotal role of RNA-binding E3 ubiquitin ligase MEX3C in RIG-I-mediated antiviral innate immunity. Proc Natl Acad Sci U S A.

[85] Lamothe B, Besse A, Campos AD, Webster WK, Wu H, Darnay BG (2007). Site-specific Lys-63-linked tumor necrosis factor receptor-associated factor 6 auto-ubiquitination is a critical determinant of I kappa B kinase activation. J Biol Chem.

[86] Li F, Li Y, Liang H, Xu T, Kong Y, Huang M, Xiao J, Chen X, Xia H, Wu Y, Zhou Z, Guo X, Hu C, Yang C, Cheng X, Chen C, Qi X (2018). HECTD3 mediates TRAF3 polyubiquitination and type I interferon induction during bacterial infection. J Clin Invest.

[87] Li S, Wang L, Berman M, Kong YY, Dorf ME (2011). Mapping a dynamic innate immunity protein interaction network regulating type I interferon production. Immunity.

[88] Li S, Zheng H, Mao AP, Zhong B, Li Y, Liu Y, Gao Y, Ran Y, Tien P, Shu HB (2010). Regulation of virus-triggered signaling by OTUB1- and OTUB2-mediated deubiquitination of TRAF3 and TRAF6. J Biol Chem.

[89] Li X, Leung S, Burns C, Stark GR (1998). Cooperative binding of Stat1-2 heterodimers and ISGF3 to tandem DNA elements. Biochimie.

[90] Li Z, Qu X, Liu X, Huan C, Wang H, Zhao Z, Yang X, Hua S, Zhang W (2020). GBP5 is an interferon-induced inhibitor of respiratory syncytial virus. J Virol.

[91] Liang J, Saad Y, Lei T, Wang J, Qi D, Yang Q, Kolattukudy PE, Fu M (2010). MCP-induced protein 1 deubiquitinates TRAF proteins and negatively regulates JNK and NF-kappaB signaling. J Exp Med.

[92] Ling Z, Tran KC, Teng MN (2009). Human respiratory syncytial virus nonstructural protein NS2 antagonizes the activation of beta interferon transcription by interacting with RIG-I. J Virol.

[93] Liu B, Zhang M, Chu H, Zhang H, Wu H, Song G, Wang P, Zhao K, Hou J, Wang X, Zhang L, Gao C (2017). The ubiquitin E3 ligase TRIM31 promotes aggregation and activation of the signaling adaptor MAVS through Lys63-linked polyubiquitination. Nat Immunol.

[94] Liu C, Huang S, Wang X, Wen M, Zheng J, Wang W, Fu Y, Tian S, Li L, Li Z, Wang X (2019). The Otubain YOD1 suppresses aggregation and activation of the signaling adaptor MAVS through Lys63-Linked Deubiquitination. J Immunol.

[95] Liu D, Chen Q, Zhu H, Gong L, Huang Y, Li S, Yue C, Wu K, Wu Y, Zhang W, Huang G, Zhang L, Pu S, Wang D (2018). Association of respiratory syncytial virus toll-like receptor 3-mediated immune response with COPD exacerbation frequency. Inflammation.

[96] Liu P, Jamaluddin M, Li K, Garofalo RP, Casola A, Brasier AR (2007). Retinoic acid-inducible gene I mediates early antiviral response and Toll-like receptor 3 expression in respiratory syncytial virus-infected airway epithelial cells. J Virol.

[97] Liu P, Li K, Garofalo RP, Brasier AR (2008). Respiratory syncytial virus induces RelA release from cytoplasmic 100-kDa NF-kappa B2 complexes via a novel retinoic acid-inducible gene-I{middle dot}NF- kappa B-inducing kinase signaling pathway. J Biol Chem.

[98] Liu P, Lu M, Tian B, Li K, Garofalo RP, Prusak D, Wood TG, Brasier AR (2009). Expression of an IKKgamma splice variant determines IRF3 and canonical NF-kappaB pathway utilization in ssRNA virus infection. PLoS ONE.

[99] Liu S, Cai X, Wu J, Cong Q, Chen X, Li T, Du F, Ren J, Wu YT, Grishin NV, Chen ZJ (2015). Phosphorylation of innate immune adaptor proteins MAVS, STING, and TRIF induces IRF3 activation. Science.

[100] Liu S, Chen J, Cai X, Wu J, Chen X, Wu YT, Sun L, Chen ZJ (2013). MAVS recruits multiple ubiquitin E3 ligases to activate antiviral signaling cascades. Elife.

[101] Liu T, Zang N, Zhou N, Li W, Xie X, Deng Y, Ren L, Long X, Li S, Zhou L, Zhao X, Tu W, Wang L, Tan B, Liu E (2014). Resveratrol inhibits the TRIF-dependent pathway by upregulating sterile alpha and armadillo motif protein, contributing to anti-inflammatory effects after respiratory syncytial virus infection. J Virol.

[102] Liu Z, Wu C, Pan Y, Liu H, Wang X, Yang Y, Gu M, Zhang Y, Wang X (2019). NDR2 promotes the antiviral immune response via facilitating TRIM25-mediated RIG-I activation in macrophages. Sci Adv.

[103] Lo MS, Brazas RM, Holtzman MJ (2005). Respiratory syncytial virus nonstructural proteins NS1 and NS2 mediate inhibition of Stat2 expression and alpha/beta interferon responsiveness. J Virol.

[104] Loo YM, Gale M (2011). Immune signaling by RIG-I-like receptors. Immunity.

[105] Maelfait J, Beyaert R (2012). Emerging role of ubiquitination in antiviral RIG-I signaling. Microbiol Mol Biol Rev.

[106] Makris S, Bajorek M, Culley FJ, Goritzka M, Johansson C (2016). Alveolar macrophages can control respiratory syncytial virus infection in the absence of type i interferons. J Innate Immun.

[107] Marr N, Turvey SE (2012). Role of human TLR4 in respiratory syncytial virus-induced NF-kappaB activation, viral entry and replication. Innate Immun.

[108] Martin-Vicente M, Gonzalez-Sanz R, Cuesta I, Monzon S, Resino S, Martinez I (2020). Downregulation of A20 expression increases the immune response and apoptosis and reduces virus production in cells infected by the human respiratory syncytial virus. Vaccines (Basel).

[109] Martin-Vicente M, Medrano LM, Resino S, Garcia-Sastre A, Martinez I (2017). TRIM25 in the regulation of the antiviral innate immunity. Front Immunol.

[110] Martin-Vicente M, Resino S, Martinez I (2019). siRNA-mediated simultaneous regulation of the cellular innate immune response and human respiratory syncytial virus replication. Biomolecules.

[111] Martinez I, Lombardia L, Garcia-Barreno B, Dominguez O, Melero JA (2007). Distinct gene subsets are induced at different time points after human respiratory syncytial virus infection of A549 cells. J Gen Virol.

[112] Massa PE, Li X, Hanidu A, Siamas J, Pariali M, Pareja J, Savitt AG, Catron KM, Li J, Marcu KB (2005). Gene expression profiling in conjunction with physiological rescues of IKKalpha-null cells with wild type or mutant IKKalpha reveals distinct classes of IKKalpha/NF-kappaB-dependent genes. J Biol Chem.

[113] Mastronarde JG, He B, Monick MM, Mukaida N, Matsushima K, Hunninghake GW (1996). Induction of interleukin (IL)-8 gene expression by respiratory syncytial virus involves activation of nuclear factor (NF)-kappa B and NF-IL-6. J Infect Dis.

[114] McCutcheon KM, Jordan R, Mawhorter ME, Noton SL, Powers JG, Fearns R, Cihlar T, Perron M (2016). The interferon type I/III response to respiratory syncytial virus infection in airway epithelial cells can be attenuated or amplified by antiviral treatment. J Virol.

[115] McDonald JU, Kaforou M, Clare S, Hale C, Ivanova M, Huntley D, Dorner M, Wright VJ, Levin M, Martinon-Torres F, Herberg JA, Tregoning JS (2016). A simple screening approach to prioritize genes for functional analysis identifies a role for interferon regulatory factor 7 in the control of respiratory syncytial virus disease. mSystems.

[116] McDowell GS, Philpott A (2013). Non-canonical ubiquitylation: mechanisms and consequences. Int J Biochem Cell Biol.

[117] McIntosh K (1978). Interferon in nasal secretions from infants with viral respiratory tract infections. J Pediatr.

[118] Moreno-Solis G, Torres-Borrego J, de la Torre-Aguilar MJ, Fernandez-Gutierrez F, Llorente-Cantarero FJ, Perez-Navero JL (2015). Analysis of the local and systemic inflammatory response in hospitalized infants with respiratory syncitial virus bronchiolitis. Allergol Immunopathol (Madr).

[119] Nair H, Nokes DJ, Gessner BD, Dherani M, Madhi SA, Singleton RJ, O'Brien KL, Roca A, Wright PF, Bruce N, Chandran A, Theodoratou E, Sutanto A, Sedyaningsih ER, Ngama M, Munywoki PK, Kartasasmita C, Simoes EA, Rudan I, Weber MW, Campbell H (2010). Global burden of acute lower respiratory infections due to respiratory syncytial virus in young children: a systematic review and meta-analysis. Lancet.

[120] Nakhaei P, Mesplede T, Solis M, Sun Q, Zhao T, Yang L, Chuang TH, Ware CF, Lin R, Hiscott J (2009). The E3 ubiquitin ligase Triad3A negatively regulates the RIG-I/MAVS signaling pathway by targeting TRAF3 for degradation. PLoS Pathog.

[121] Oganesyan G, Saha SK, Guo B, He JQ, Shahangian A, Zarnegar B, Perry A, Cheng G (2006). Critical role of TRAF3 in the Toll-like receptor-dependent and -independent antiviral response. Nature.

[122] Oshiumi H, Matsumoto M, Hatakeyama S, Seya T (2009). Riplet/RNF135, a RING finger protein, ubiquitinates RIG-I to promote interferon-beta induction during the early phase of viral infection. J Biol Chem.

[123] Oshiumi H, Matsumoto M, Seya T (2012). Ubiquitin-mediated modulation of the cytoplasmic viral RNA sensor RIG-I. J Biochem.

[124] Oshiumi H, Miyashita M, Inoue N, Okabe M, Matsumoto M, Seya T (2010). The ubiquitin ligase Riplet is essential for RIG-I-dependent innate immune responses to RNA virus infection. Cell Host Microbe.

[125] Oshiumi H, Miyashita M, Matsumoto M, Seya T (2013). A distinct role of Riplet-mediated K63-Linked polyubiquitination of the RIG-I repressor domain in human antiviral innate immune responses. PLoS Pathog.

[126] Pan Y, Li R, Meng JL, Mao HT, Zhang Y, Zhang J (2014). Smurf2 negatively modulates RIG-I-dependent antiviral response by targeting VISA/MAVS for ubiquitination and degradation. J Immunol.

[127] Parvatiyar K, Barber GN, Harhaj EW (2010). TAX1BP1 and A20 inhibit antiviral signaling by targeting TBK1-IKKi kinases. J Biol Chem.

[128] Pattabhi S, Wilkins CR, Dong R, Knoll ML, Posakony J, Kaiser S, Mire CE, Wang ML, Ireton RC, Geisbert TW, Bedard KM, Iadonato SP, Loo YM, Gale M (2015). Targeting innate immunity for antiviral therapy through small molecule agonists of the RLR Pathway. J Virol.

[129] Paulsen M, Varese A, Pinpathomrat N, Kirsebom FCM, Paulsen M, Johansson C (2020). MAVS deficiency is associated with a reduced t cell response upon secondary RSV infection in mice. Front Immunol.

[130] Paz S, Sun Q, Nakhaei P, Romieu-Mourez R, Goubau D, Julkunen I, Lin R, Hiscott J (2006). Induction of IRF-3 and IRF-7 phosphorylation following activation of the RIG-I pathway. Cell Mol Biol.

[131] Paz S, Vilasco M, Werden SJ, Arguello M, Joseph-Pillai D, Zhao T, Nguyen TL, Sun Q, Meurs EF, Lin R, Hiscott J (2011). A functional C-terminal TRAF3-binding site in MAVS participates in positive and negative regulation of the IFN antiviral response. Cell Res.

[132] Pei J, Wagner ND, Zou AJ, Chatterjee S, Borek D, Cole AR, Kim PJ, Basler CF, Otwinowski Z, Gross ML, Amarasinghe GK, Leung DW (2021). Structural basis for IFN antagonism by human respiratory syncytial virus nonstructural protein 2. Proc Natl Acad Sci U S A.

[133] Peisley A, Wu B, Xu H, Chen ZJ, Hur S (2014). Structural basis for ubiquitin-mediated antiviral signal activation by RIG-I. Nature.

[134] Pickart CM, Eddins MJ (2004). Ubiquitin: structures, functions, mechanisms. Biochim Biophys Acta.

[135] Pickles RJ, DeVincenzo JP (2015). Respiratory syncytial virus (RSV) and its propensity for causing bronchiolitis. J Pathol.

[136] Pyle CJ, Uwadiae FI, Swieboda DP, Harker JA (2017). Early IL-6 signalling promotes IL-27 dependent maturation of regulatory T cells in the lungs and resolution of viral immunopathology. PLoS Pathog.

[137] Rajsbaum R, Albrecht RA, Wang MK, Maharaj NP, Versteeg GA, Nistal-Villan E, Garcia-Sastre A, Gack MU (2012). Species-specific inhibition of RIG-I ubiquitination and IFN induction by the influenza A virus NS1 protein. PLoS Pathog.

[138] Ramaswamy M, Shi L, Monick MM, Hunninghake GW, Look DC (2004). Specific inhibition of type I interferon signal transduction by respiratory syncytial virus. Am J Respir Cell Mol Biol.

[139] Reimers K, Buchholz K, Werchau H (2005). Respiratory syncytial virus M2–1 protein induces the activation of nuclear factor kappa B. Virology.

[140] Ren J, Liu T, Pang L, Li K, Garofalo RP, Casola A, Bao X (2011). A novel mechanism for the inhibition of interferon regulatory factor-3-dependent gene expression by human respiratory syncytial virus NS1 protein. J Gen Virol.

[141] Rudd BD, Burstein E, Duckett CS, Li X, Lukacs NW (2005). Differential role for TLR3 in respiratory syncytial virus-induced chemokine expression. J Virol.

[142] Saha SK, Pietras EM, He JQ, Kang JR, Liu SY, Oganesyan G, Shahangian A, Zarnegar B, Shiba TL, Wang Y, Cheng G (2006). Regulation of antiviral responses by a direct and specific interaction between TRAF3 and Cardif. EMBO J.

[143] Saito T, Hirai R, Loo YM, Owen D, Johnson CL, Sinha SC, Akira S, Fujita T, Gale M (2007). Regulation of innate antiviral defenses through a shared repressor domain in RIG-I and LGP2. Proc Natl Acad Sci U S A.

[144] Saitoh T, Yamamoto M, Miyagishi M, Taira K, Nakanishi M, Fujita T, Akira S, Yamamoto N, Yamaoka S (2005). A20 is a negative regulator of IFN regulatory factor 3 signaling. J Immunol.

[145] Sanchez JG, Chiang JJ, Sparrer KMJ, Alam SL, Chi M, Roganowicz MD, Sankaran B, Gack MU, Pornillos O (2016). Mechanism of TRIM25 Catalytic Activation in the Antiviral RIG-I Pathway. Cell Rep.

[146] Scagnolari C, Midulla F, Pierangeli A, Moretti C, Bonci E, Berardi R, De Angelis D, Selvaggi C, Di Marco P, Girardi E, Antonelli G (2009). Gene expression of nucleic acid-sensing pattern recognition receptors in children hospitalized for respiratory syncytial virus-associated acute bronchiolitis. Clin Vaccine Immunol.

[147] Schlender J, Bossert B, Buchholz U, Conzelmann KK (2000). Bovine respiratory syncytial virus nonstructural proteins NS1 and NS2 cooperatively antagonize alpha/beta interferon-induced antiviral response. J Virol.

[148] Sharma S, tenOever BR, Grandvaux N, Zhou GP, Lin R, Hiscott J (2003). Triggering the interferon antiviral response through an IKK-related pathway. Science.

[149] Shembade N, Ma A, Harhaj EW (2010). Inhibition of NF-kappaB signaling by A20 through disruption of ubiquitin enzyme complexes. Science.

[150] Shi T, McAllister DA, OBrien KL, Simoes EAF, Madhi SA, Gessner BD, Polack FP, Balsells E, Acacio S, Aguayo C, Alassani I, Ali A, Antonio M, Awasthi S, Awori JO, Azziz-Baumgartner E, Baggett HC, Baillie VL, Balmaseda A, Barahona A, Basnet S, Bassat Q, Basualdo W, Bigogo G, Bont L, Breiman RF, Brooks WA, Broor S, Bruce N, Bruden D, Buchy P, Campbell S, Carosone-Link P, Chadha M, Chipeta J, Chou M, Clara W, Cohen C, de Cuellar E, Dang DA, Dash-Yandag B, Deloria-Knoll M, Dherani M, Eap T, Ebruke BE, Echavarria M, de Freitas CC, Fasce RA, Feikin DR, Feng L, Gentile A, Gordon A, Goswami D, Goyet S, Groome M, Halasa N, Hirve S, Homaira N, Howie SRC, Jara J, Jroundi I, Kartasasmita CB, Khuri-Bulos N, Kotloff KL, Krishnan A, Libster R, Lopez O, Lucero MG, Lucion F, Lupisan SP, Marcone DN, McCracken JP, Mejia M, Moisi JC, Montgomery JM, Moore DP, Moraleda C, Moyes J, Munywoki P, Mutyara K, Nicol MP, Nokes DJ, Nymadawa P, CostaOliveira MT, Oshitani H, Pandey N, Paranhos-Baccala G, Phillips LN, Picot VS, Rahman M, Rakoto-Andrianarivelo M, Rasmussen ZA, Rath BA, Robinson A, Romero C, Russomando G, Salimi V, Sawatwong P, Scheltema N, Schweiger B, Scott JAG, Seidenberg P, Shen K, Singleton R, Sotomayor V, Strand TA, Sutanto A, Sylla M, Tapia MD, Thamthitiwat S, Thomas ED, Tokarz R, Turner C, Venter M, Waicharoen S, Wang J, Watthanaworawit W, Yoshida LM, Yu H, Zar HJ, Campbell H, Nair H. Global, regional, and national disease burden estimates of acute lower respiratory infections due to respiratory syncytial virus in young children in 2015: a systematic review and modelling study. Lancet. 2017; 390(6):946–958. 10.1016/S0140-6736(17)30938-8 PMC5592248 28689664

[151] Shin C, Ito Y, Ichikawa S, Tokunaga M, Sakata-Sogawa K, Tanaka T (2017). MKRN2 is a novel ubiquitin E3 ligase for the p65 subunit of NF-kappaB and negatively regulates inflammatory responses. Sci Rep.

[152] Song G, Liu B, Li Z, Wu H, Wang P, Zhao K, Jiang G, Zhang L, Gao C (2016). E3 ubiquitin ligase RNF128 promotes innate antiviral immunity through K63-linked ubiquitination of TBK1. Nat Immunol.

[153] Song Y, Lai L, Chong Z, He J, Zhang Y, Xue Y, Xie Y, Chen S, Dong P, Chen L, Chen Z, Dai F, Wan X, Xiao P, Cao X, Liu Y, Wang Q (2017). E3 ligase FBXW7 is critical for RIG-I stabilization during antiviral responses. Nat Commun.

[154] Spann KM, Tran KC, Chi B, Rabin RL, Collins PL (2004). Suppression of the induction of alpha, beta, and lambda interferons by the NS1 and NS2 proteins of human respiratory syncytial virus in human epithelial cells and macrophages [corrected]. J Virol.

[155] Spann KM, Tran KC, Collins PL (2005). Effects of nonstructural proteins NS1 and NS2 of human respiratory syncytial virus on interferon regulatory factor 3, NF-kappaB, and proinflammatory cytokines. J Virol.

[156] Stier MT, Goleniewska K, Cephus JY, Newcomb DC, Sherrill TP, Boyd KL, Bloodworth MH, Moore ML, Chen K, Kolls JK, Peebles RS (2017). STAT1 represses cytokine-producing group 2 and group 3 innate lymphoid cells during viral infection. J Immunol.

[157] Sun L, Deng L, Ea CK, Xia ZP, Chen ZJ (2004). The TRAF6 ubiquitin ligase and TAK1 kinase mediate IKK activation by BCL10 and MALT1 in T lymphocytes. Mol Cell.

[158] Sun Y, Lopez CB (2017). The innate immune response to RSV: Advances in our understanding of critical viral and host factors. Vaccine.

[159] Swedan S, Andrews J, Majumdar T, Musiyenko A, Barik S (2011). Multiple functional domains and complexes of the two nonstructural proteins of human respiratory syncytial virus contribute to interferon suppression and cellular location. J Virol.

[160] Swedan S, Musiyenko A, Barik S (2009). Respiratory syncytial virus nonstructural proteins decrease levels of multiple members of the cellular interferon pathways. J Virol.

[161] Takahasi K, Yoneyama M, Nishihori T, Hirai R, Kumeta H, Narita R, Gale M, Inagaki F, Fujita T (2008). Nonself RNA-sensing mechanism of RIG-I helicase and activation of antiviral immune responses. Mol Cell.

[162] Takeuchi O, Akira S (2010). Pattern recognition receptors and inflammation. Cell.

[163] Tanaka T, Grusby MJ, Kaisho T (2007). PDLIM2-mediated termination of transcription factor NF-kappaB activation by intranuclear sequestration and degradation of the p65 subunit. Nat Immunol.

[164] Thomas KW, Monick MM, Staber JM, Yarovinsky T, Carter AB, Hunninghake GW (2002). Respiratory syncytial virus inhibits apoptosis and induces NF-kappa B activity through a phosphatidylinositol 3-kinase-dependent pathway. J Biol Chem.

[165] Thomas LH, Friedland JS, Sharland M, Becker S (1998). Respiratory syncytial virus-induced RANTES production from human bronchial epithelial cells is dependent on nuclear factor-kappa B nuclear binding and is inhibited by adenovirus-mediated expression of inhibitor of kappa B alpha. J Immunol.

[166] Thornburg NJ, Hayward SL, Crowe JE (2012). Respiratory syncytial virus regulates human microRNAs by using mechanisms involving beta interferon and NF-kappaB. MBio.

[167] Tian B, Yang J, Zhao Y, Ivanciuc T, Sun H, Garofalo RP, Brasier AR (2017). BRD4 Couples NF-kappaB/RelA with airway inflammation and the IRF-RIG-I amplification loop in respiratory syncytial virus infection. J Virol.

[168] Tian B, Zhang Y, Luxon BA, Garofalo RP, Casola A, Sinha M, Brasier AR (2002). Identification of NF-kappaB-dependent gene networks in respiratory syncytial virus-infected cells. J Virol.

[169] Tokunaga F, Sakata S, Saeki Y, Satomi Y, Kirisako T, Kamei K, Nakagawa T, Kato M, Murata S, Yamaoka S, Yamamoto M, Akira S, Takao T, Tanaka K, Iwai K (2009). Involvement of linear polyubiquitylation of NEMO in NF-kappaB activation. Nat Cell Biol.

[170] Wang C, Chen T, Zhang J, Yang M, Li N, Xu X, Cao X (2009). The E3 ubiquitin ligase Nrdp1 'preferentially' promotes TLR-mediated production of type I interferon. Nat Immunol.

[171] Wang C, Deng L, Hong M, Akkaraju GR, Inoue J, Chen ZJ (2001). TAK1 is a ubiquitin-dependent kinase of MKK and IKK. Nature.

[172] Wang L, Zhao W, Zhang M, Wang P, Zhao K, Zhao X, Yang S, Gao C (2013). USP4 positively regulates RIG-I-mediated antiviral response through deubiquitination and stabilization of RIG-I. J Virol.

[173] Wang P, Zhao W, Zhao K, Zhang L, Gao C (2015). TRIM26 negatively regulates interferon-beta production and antiviral response through polyubiquitination and degradation of nuclear IRF3. PLoS Pathog.

[174] Wang W, Jiang M, Liu S, Zhang S, Liu W, Ma Y, Zhang L, Zhang J, Cao X (2016). RNF122 suppresses antiviral type I interferon production by targeting RIG-I CARDs to mediate RIG-I degradation. Proc Natl Acad Sci U S A.

[175] Wang Y, Chen Y, Lin Y, Quan Y, Xiao X, Zhang R (2021). TRIM22 inhibits respiratory syncytial virus replication by targeting JAK-STAT1/2 signaling. J Med Virol.

[176] Wang Y, Tong X, Ye X (2012). Ndfip1 negatively regulates RIG-I-dependent immune signaling by enhancing E3 ligase Smurf1-mediated MAVS degradation. J Immunol.

[177] Xing J, Weng L, Yuan B, Wang Z, Jia L, Jin R, Lu H, Li XC, Liu YJ, Zhang Z (2016). Identification of a role for TRIM29 in the control of innate immunity in the respiratory tract. Nat Immunol.

[178] Xing J, Zhang A, Minze LJ, Li XC, Zhang Z (2018). TRIM29 Negatively Regulates the Type I IFN production in response to RNA Virus. J Immunol.

[179] Xu LG, Wang YY, Han KJ, Li LY, Zhai Z, Shu HB (2005). VISA is an adapter protein required for virus-triggered IFN-beta signaling. Mol Cell.

[180] Xue B, Li H, Guo M, Wang J, Xu Y, Zou X, Deng R, Li G, Zhu H (2018). TRIM21 Promotes Innate Immune Response to RNA Viral Infection through Lys27-Linked Polyubiquitination of MAVS. J Virol.

[181] Yan J, Li Q, Mao AP, Hu MM, Shu HB (2014). TRIM4 modulates type I interferon induction and cellular antiviral response by targeting RIG-I for K63-linked ubiquitination. J Mol Cell Biol.

[182] Ye Y, Rape M (2009). Building ubiquitin chains: E2 enzymes at work. Nat Rev Mol Cell Biol.

[183] Yoboua F, Martel A, Duval A, Mukawera E, Grandvaux N (2010). Respiratory syncytial virus-mediated NF-kappa B p65 phosphorylation at serine 536 is dependent on RIG-I, TRAF6, and IKK beta. J Virol.

[184] Yoneyama M, Kikuchi M, Matsumoto K, Imaizumi T, Miyagishi M, Taira K, Foy E, Loo YM, Gale M, Akira S, Yonehara S, Kato A, Fujita T (2005). Shared and unique functions of the DExD/H-box helicases RIG-I, MDA5, and LGP2 in antiviral innate immunity. J Immunol.

[185] Yoneyama M, Kikuchi M, Natsukawa T, Shinobu N, Imaizumi T, Miyagishi M, Taira K, Akira S, Fujita T (2004). The RNA helicase RIG-I has an essential function in double-stranded RNA-induced innate antiviral responses. Nat Immunol.

[186] Yoo YS, Park YY, Kim JH, Cho H, Kim SH, Lee HS, Kim TH, Sun KY, Lee Y, Kim CJ, Jung JU, Lee JS, Cho H (2015). The mitochondrial ubiquitin ligase MARCH5 resolves MAVS aggregates during antiviral signalling. Nat Commun.

[187] You F, Sun H, Zhou X, Sun W, Liang S, Zhai Z, Jiang Z (2009). PCBP2 mediates degradation of the adaptor MAVS via the HECT ubiquitin ligase AIP4. Nat Immunol.

[188] Yu Y, Hayward GS (2010). The ubiquitin E3 ligase RAUL negatively regulates type i interferon through ubiquitination of the transcription factors IRF7 and IRF3. Immunity.

[189] Zeng W, Sun L, Jiang X, Chen X, Hou F, Adhikari A, Xu M, Chen ZJ (2010). Reconstitution of the RIG-I pathway reveals a signaling role of unanchored polyubiquitin chains in innate immunity. Cell.

[190] Zhang M, Tian Y, Wang RP, Gao D, Zhang Y, Diao FC, Chen DY, Zhai ZH, Shu HB (2008). Negative feedback regulation of cellular antiviral signaling by RBCK1-mediated degradation of IRF3. Cell Res.

[191] Zhang M, Wang L, Zhao X, Zhao K, Meng H, Zhao W, Gao C (2012). TRAF-interacting protein (TRIP) negatively regulates IFN-beta production and antiviral response by promoting proteasomal degradation of TANK-binding kinase 1. J Exp Med.

[192] Zhang Y, Luxon BA, Casola A, Garofalo RP, Jamaluddin M, Brasier AR (2001). Expression of respiratory syncytial virus-induced chemokine gene networks in lower airway epithelial cells revealed by cDNA microarrays. J Virol.

[193] Zhao C, Jia M, Song H, Yu Z, Wang W, Li Q, Zhang L, Zhao W, Cao X (2017). The E3 Ubiquitin Ligase TRIM40 attenuates antiviral immune responses by targeting MDA5 and RIG-I. Cell Rep.

[194] Zhao T, Yang L, Sun Q, Arguello M, Ballard DW, Hiscott J, Lin R (2007). The NEMO adaptor bridges the nuclear factor-kappaB and interferon regulatory factor signaling pathways. Nat Immunol.

[195] Zhao W, Wang L, Zhang M, Yuan C, Gao C (2012). E3 ubiquitin ligase tripartite motif 38 negatively regulates TLR-mediated immune responses by proteasomal degradation of TNF receptor-associated factor 6 in macrophages. J Immunol.

[196] Zhao X, Zhu H, Yu J, Li H, Ge J, Chen W (2016). c-Cbl-mediated ubiquitination of IRF3 negatively regulates IFN-beta production and cellular antiviral response. Cell Signal.

[197] Zheng N, Shabek N (2017). Ubiquitin ligases: structure, function, and regulation. Annu Rev Biochem.

[198] Zheng Q, Hou J, Zhou Y, Yang Y, Xie B, Cao X (2015). Siglec1 suppresses antiviral innate immune response by inducing TBK1 degradation via the ubiquitin ligase TRIM27. Cell Res.

[199] Zhong B, Zhang Y, Tan B, Liu TT, Wang YY, Shu HB (2010). The E3 ubiquitin ligase RNF5 targets virus-induced signaling adaptor for ubiquitination and degradation. J Immunol.

[200] Zinngrebe J, Montinaro A, Peltzer N, Walczak H (2014). Ubiquitin in the immune system. EMBO Rep.

[201] Zotti T, Uva A, Ferravante A, Vessichelli M, Scudiero I, Ceccarelli M, Vito P, Stilo R (2011). TRAF7 protein promotes Lys-29-linked polyubiquitination of IkappaB kinase (IKKgamma)/NF-kappaB essential modulator (NEMO) and p65/RelA protein and represses NF-kappaB activation. J Biol Chem.

[202] Zuo Y, Feng Q, Jin L, Huang F, Miao Y, Liu J, Xu Y, Chen X, Zhang H, Guo T, Yuan Y, Zhang L, Wang J, Zheng H (2020). Regulation of the linear ubiquitination of STAT1 controls antiviral interferon signaling. Nat Commun.

